# Revealing the Role of Hydrogen in Highly Efficient Ag-Substituted CZTSSe Photovoltaic Devices: Photoelectric Properties Modulation and Defect Passivation

**DOI:** 10.1007/s40820-024-01574-3

**Published:** 2024-12-03

**Authors:** Xiaoyue Zhao, Jingru Li, Chenyang Hu, Yafang Qi, Zhengji Zhou, Dongxing Kou, Wenhui Zhou, Shengjie Yuan, Sixin Wu

**Affiliations:** The Key Laboratory for Special Functional Materials of MOE, School of Nanoscience and Materials Engineering, National and Local Joint Engineering Research Center for High-Efficiency Display and Lighting Technology, Collaborative Innovation Center of Nano Functional Materials and Applications, Henan University, Kaifeng, 475004 People’s Republic of China

**Keywords:** CZTSSe, Ag/H co-doping, Photoelectric properties modulation, Defect passivation, Non-radiative recombination

## Abstract

The presence of Sn_Zn_-related defects in Cu_2_ZnSn(S,Se)_4_ (CZTSSe) absorber results in large irreversible energy loss and extra irreversible electron–hole non-radiative recombination, thus hindering the efficiency enhancement of CZTSSe devices. Although the incorporation of Ag in CZTSSe can effectively suppress the Sn_Zn_-related defects and significantly improve the resulting cell performance, an excellent efficiency has not been achieved to date primarily owing to the poor electrical-conductivity and the low carrier density of the CZTSSe film induced by Ag substitution. Herein, this study exquisitely devises an Ag/H co-doping strategy in CZTSSe absorber via Ag substitution programs followed by hydrogen-plasma treatment procedure to suppress Sn_Zn_ defects for achieving efficient CZTSSe devices. In-depth investigation results demonstrate that the incorporation of H in Ag-based CZTSSe absorber is expected to improve the poor electrical-conductivity and the low carrier density caused by Ag substitution. Importantly, the C=O and O–H functional groups induced by hydrogen incorporation, serving as an electron donor, can interact with under-coordinated cations in CZTSSe material, effectively passivating the Sn_Zn_-related defects. Consequently, the incorporation of an appropriate amount of Ag/H in CZTSSe mitigates carrier non-radiative recombination, prolongs minority carrier lifetime, and thus yields a champion efficiency of 14.74%, showing its promising application in kesterite-based CZTSSe devices.

**Supplementary Information:**

The online version contains supplementary material available at 10.1007/s40820-024-01574-3.

## Highlights

- The Ag/H co-doping strategy has been proposed to promote the performance of Cu_2_ZnSn(S,Se)_4_ (CZTSSe) devices.
- The incorporation of H in Ag-based CZTSSe photovoltaic absorber is expected to improve the poor electrical conductivity and the low carrier density caused by Ag substitution
- Benefiting from the synergism of the excellent defect passivation effect and photoelectric properties complementary effect enabled by Ag/H co-doping, a champion device with 14.74% efficiency is achieved

**Supplementary Information:**

The online version contains supplementary material available at 10.1007/s40820-024-01574-3.

## Graphical abstract

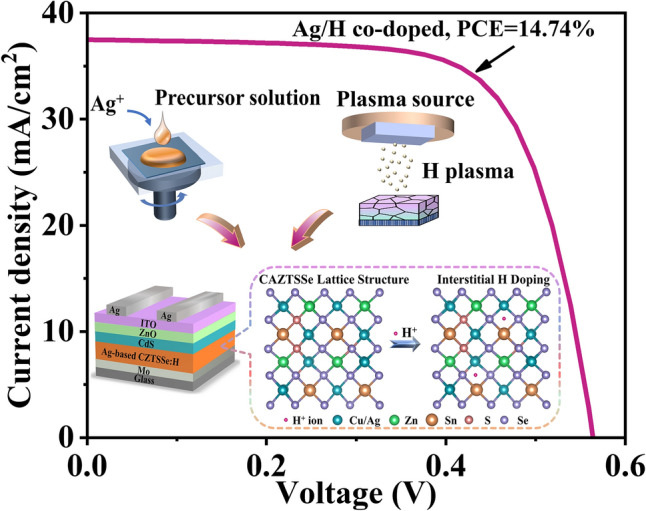

## Introduction

Kesterite Cu_2_ZnSn(S,Se)_4_ (CZTSSe) solar cells have emerged as a promising candidates for the next-generation thin-film photovoltaic technologies due to its incomparable advantages, such as low cost, high absorption coefficient, adjustable bandgap, and high theoretical conversion efficiency [1–5]. In the past decade, the certified power conversion efficiency (PCE) of CZTSSe device had made incredible progress, soaring from 12.6% to over 15% [6, 7]. Even so, compared with its predecessors, including CIGS (23.6%), cadmium telluride (22.6%), and silicon (26.81%) solar cells [7–9], there is still a great gap. Therefore, it is imperative to explore the internal loss mechanism that affects the improvement of cell performance to chase the high-performance CZTSSe devices.

It is generally recognized that the Shockley-Queisser (S-Q) theoretical limit efficiency of CZTSSe solar cells is 32.8%, which is a prediction of theoretical sunlight-to-electricity conversion efficiency of a single-junction solar cell based on the principle of detailed balance [10]. In reality, however, few materials approach this radiative limit. For single-junction CZTSe (CZTS) solar cells, its intrinsic limit efficiency is only 20.3% (20.9%), due to the presence of large irreversible energy loss and extra irreversible electron–hole non-radiative recombination [11, 12]. In this regard, suppressing the non-radiative recombination loss is imperative for the CZTSSe device performance to reach S-Q theoretical limit. Encouragingly, recent theoretical calculations and experimental studies have proposed that the deep defects, such as Sn_Zn_ donor and associated compensated defect clusters, in CZTSSe absorber bulk are often responsible for carrier trapping and non-radiative recombination owing to its high concentration (> 10^14^ cm^−3^), which could result in a shorter carrier lifetime and a lower open-circuit voltage (*V*_OC_), limiting the performance of resulting devices [13–15].

In an attempt to address these issues, a number of studies have been conducted to suppresses the formation of Sn_Zn_-related defects [1, 4, 16–21], and thus, to radically promote the *V*_OC_ and PCE of CZTSSe solar cells. Some studies have shown that the proper isoelectronic cation substitution in the host CZTSSe crystal lattice is an effective method to address this issue [17, 18]. Other studies highlight the importance of local chemical environment engineering in suppressing detrimental intrinsic defect (i.e., Sn_Zn_) in CZTSSe devices [20, 21]. In particular, theoretical studies strongly indicate that substitute Cu with Ag for the formation of Ag_2_ZnSnSe_4_ (AZTSe) semiconductor should show much lower non-radiative recombination, implying that AZTSe is a promising material as a photovoltaic absorber [22]. Unfortunately, previous experiments have shown that the electrical conductivity of AZTSe semiconductor is always very poor, and the low carrier density cannot support an expected device performance [5, 23–25]. More importantly, the n-type behavior of AZTSe semiconductor makes the structure of CZTSSe device unsuitable [26, 27]. Therefore, it is critical to enhance the p-type nature and increase the carrier density of Ag-based kesterite materials for further optimizing the device performance. One possible strategy to enhance the p-type behavior of AZTSe materials is incorporation of hydrogen (H) to increase the hole carrier concentration [28–30]. Equally important, recent theoretical studies have demonstrated that the introduction of H in CZTSSe-based semiconductor can reduce the concentration of Sn_Zn_ defects and suppress trap-assisted non-radiative recombination more effectively [29], ultimately the intrinsic limit efficiency improved significantly from 20% to 23.7% [31, 32]. Further, in theory, if H is introduced into the AZTSe material, it is expected to reduce the concentration of Sn_Zn_ below 10^14^ cm^−3^, thereby further increasing the performance threshold to 30.8% [32].

Considering these aspects, we herein proposed an Ag/H co-doping strategy in CZTSSe photovoltaic absorber to inhibit the formation of Sn_Zn_-related defects, and thus, to promote the *V*_OC_ and PCE. In this process, Ag is incorporated into the CZTSSe absorber by partially replacing Cu to ensure the device architecture is not affected, whereas the incorporation of H in the Ag-based CZTSSe film is achieved via the hydrogen plasma (H-plasma) treatment procedure. The incorporation of Ag in CZTSSe absorber was observed to increases grain size and enhances the charge extraction. At the same time, it also leads to the decrease of electrical conductivity and carrier concentration. Taken as a supplement, the introduction of hydrogen enhances the electrical conductivity and carrier concentration in Ag-based CZTSSe film. More importantly, the Ag/H co-doping strategy can also play an effective role in passivating Sn_Zn_-related donor defects in the CZTSSe-based samples, substantially diminishing trap-assisted non-radiative recombination of charge carrier. Benefiting from the synergism of the excellent defect passivation effect and photoelectric properties complementary effect enabled by Ag/H co-doping, the champion PCE of the CZTSSe devices were enhanced from 11.94% (reference) to 12.62% (Ag-doped) and 14.74% (Ag/H co-doped), largely attributing to the 80.75 mV *V*_OC_ enhancement.

## Experimental

### Preparation of the CZTSSe Precursor Solution

Zn(CH_3_COO)_2_·2H_2_O (0.20 mol L^−1^), SnCl_4_·5H_2_O (0.17 mol L^−1^), CuCl (or CuCl and AgCl, 0.28 mol L^−1^), and SC(NH_2_)_2_ (1.33 mol L^−1^) were dissolved in 10 mL of 2-methoxyethanol to prepare a CZTSSe precursor solution. To investigate the influence of Ag content on the performance of CZTSSe devices, the precursor solution with Ag/(Ag + Cu) ratios of 0, 10, 15, and 20 wt% were prepared.

### Ag-Based CZTSSe Absorber Preparation and H-Plasma Treatment Processes

The prepared precursor solutions with different Ag contents were spin-coated on a molybdenum-coated soda-lime glass substrate (3000 r min^−1^ for 30 s) and then sintered on the hot plate (290 °C, 2 min) to remove the organic residues. The spin-coating/sintering steps were repeated 12 times until the precursor films has a targeted thickness (~1.1 μm). The as-prepared precursor films were then put into a graphite box containing selenium particles and annealed in a rapid thermal processing (RTP) furnace. After selenization at 530 °C for 10 min, the CZTSSe absorber film is finally obtained.

The Ag-based CZTSSe absorbers were then subjected to H-plasma treatment procedure in a plasma-enhanced chemical vapour deposition (PECVD) system. To investigate how different content of H influences the performance of solar cells, the CZTSSe thin films received a H-plasma treatment procedure in the gas mixture of H_2_/Ar with the concentration of H varying from 0 to 10%. Subsequently, the effects of H-plasma treatment powers (0, 20, 40, 50, 60, and 80 W) and the H-plasma treatment times (0, 10, 20, 25, 30, and 40 s) on device performance were also investigated. Next, the prepared Ag/H co-doped CZTSSe thin films were heated on a 200 °C hot plate for 10 min in a glove box with nitrogen flow to promote the diffusion of hydrogen. It should be noted that the Reference and Ag-doped absorbers were also annealed (200 °C, 10 min) in this work.

### Fabrication of CZTSSe Photovoltaic Devices

The CZTSSe solar cells were fabricated according to the standard configuration of soda-lime glass/Mo/CZTSSe/CdS/i-ZnO/ITO/Ag-grid as published previously [33, 34].

### Characterization

Time-of-flight secondary ion mass spectroscopy (TOF–SIMS), Fourier transform infrared (FT-IR) spectroscopy, X-ray photoelectron spectroscopy (XPS), scanning electron microscopy (SEM), X-ray diffraction (XRD), Raman spectroscopy, Hall measurement, Kelvin probe force microscopy (KPFM), current density–voltage (*J–V*) curve, external quantum efficiency (EQE) spectra, deep-level transient spectroscopy (DLTS), electrochemical impedance spectroscopy (EIS), transient photovoltage (TPV) spectra, transient photocurrent (TPC) spectra, photoluminescence (PL) spectroscopy and time-resolved photoluminescence (TRPL) spectroscopy, temperature-dependent dark *J–V* (*J–V–T*) profile, capacitance–voltage (*C–V*) curve, drive-level capacitance profiling (DLCP), were all carried out as in published previously [35, 36]. In addition, the electron paramagnetic resonance (EPR) spectra were measured by an Electron Paramagnetic Resonance Instrument A300-10/12 from Bruker, Germany.

## Results and Discussion

### Characterization of the CZTSSe Thin Films

**Fig. 1:**
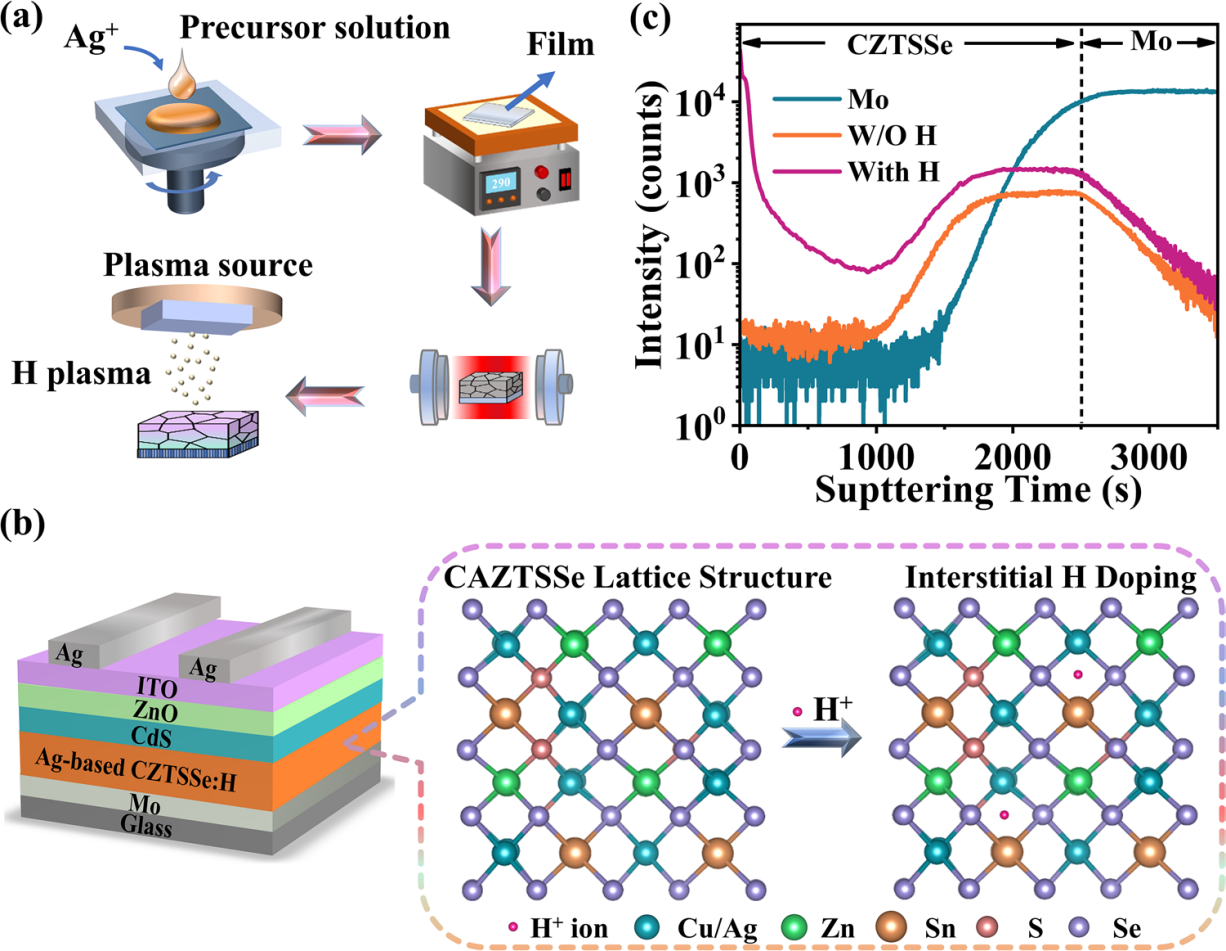
**a** Schematic description of the Ag/H co-doping procedure for the CZTSSe absorbers. **b** Schematic diagram of CZTSSe-based solar cells and the crystal model before and after H doping. **c** TOF–SIMS depth profiles of H intensities in CAZTSSe thin films without and with H-doping

In this work, we adopted the chemical-based substitution (solution) and plasma treatment methods to fabricate the Ag/H co-doped CZTSSe absorbers (Fig. 1a). Various process parameters, such as Ag content, hydrogen input (concentration), H-plasma treatment power, and H-plasma treatment time, are systematically investigated to optimize the cell performance. Figure 1b illustrates the crystal model before and after H doping, from which the actual location of hydrogen after entering the lattice can be clearly seen. The elemental depth profiles of the CAZTSSe thin films without and with H-doping were first carefully studied using time-of-flight secondary ion mass spectrometry (TOF–SIMS) analysis. From Fig. 1c, it is clear that the H-doped CAZTSSe sample exhibits a higher H signal in the whole absorber bulk in contrast to the sample without H-doping. Notably, the signal of H element in the Reference sample probably comes from the organic residues inside the precursor solution. Besides, the H element is not uniformly distributed in the absorber bulk of the H-doped CAZTSSe sample, which is attributed to the effect of the additional heat treatment. The increase of H signal in the bottom layer of the H-doped CAZTSSe sample can be explained by the organic residues inside the precursor solution and the presence of a small grain layer at the bottom layer near the Mo substrate. Besides, for the other elements, such as Cu, Ag, Zn, and Sn, both the Ag-substituted sample and Ag/H co-doped sample exhibit a similar depth profile throughout the whole CZTSSe absorbers (Fig. S1a, b). For the sake of discussion, the untreated CZTSSe sample is referred to as “Reference” sample, while the treated samples are, respectively, designated as “Ag-doped” and “Ag/H co-doped” samples in the following discussion.

**Fig. 2:**
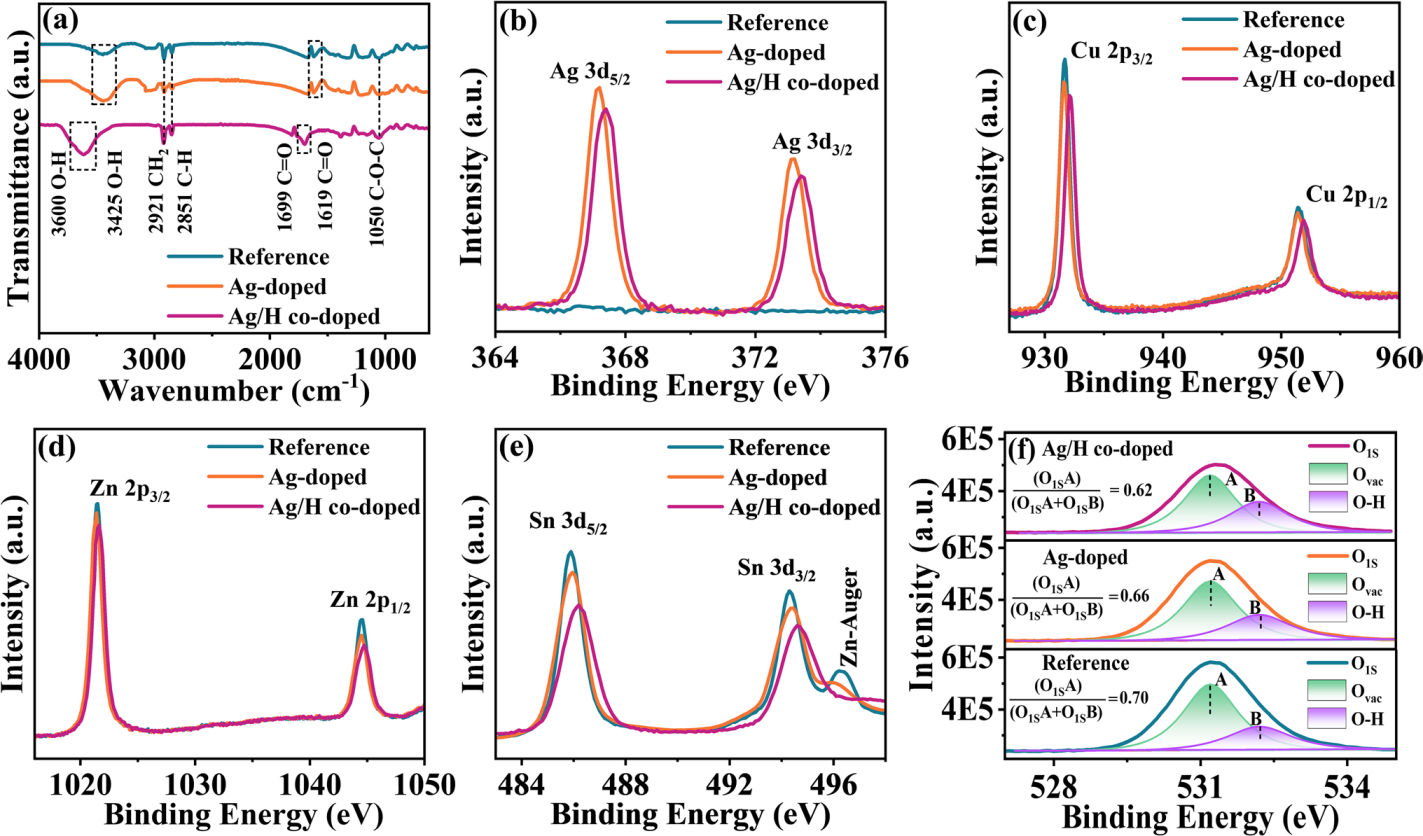
**a** FTIR spectra of the Reference, Ag-doped, and Ag/H co-doped CZTSSe absorbers. XPS spectra of **b** Ag 3*d*, **c** Cu 2*p*, **d** Zn 2*p*, **e** Sn 3*d*, and **f** fits of the XPS O 1*s* peaks for the Reference, Ag-doped, Ag/H co-doped CZTSSe thin films

To investigate the potential interactions of hydrogen and the CAZTSSe film, FTIR measurements were carried out. As illustrated in Fig. 2a, the vibrational peaks located at 3425/3600, 2921, 2851, 1619/1699, and 1050 cm^−1^ correspond to the O–H, CH_2_, C–H, C=O, and C–O–C bonds [37–40], respectively. Apparently, compared with the reference and Ag-doped CZTSSe samples, the C=O stretching vibrations peak and O–H stretching vibrations peak for the Ag/H co-doped sample shift from 1619 and 3425 cm^−1^ to 1699 and 3600 cm^−1^, respectively. Moreover, the Ag/H co-doped sample show much stronger C=O and O–H peaks than the Reference and Ag-doped CZTSSe samples. These results indicate a change in the electron cloud distribution induced by hydrogen incorporation, which may potentially serve as an electron donor [29, 30, 32], can interact with under-coordinated cations in CZTSSe material, ultimately passivates the Sn_Zn_-related defects in the final devices.

Next, we further analyzed the surface chemical environment change of the CZTSSe samples by XPS measurement, as displayed in Fig. 2b–f. As expected, the valence states of Ag, Cu, Zn, and Sn elements have not shown significant change after H-plasma treatment. It is worth noting that the positions of the XPS peaks shifted toward a high binding energy direction after H-doping, indicating a change in chemical environment [33, 41]. These shift can be ascribed to the interaction between oxygen-containing functional groups (–OH and C = O) induced by hydrogen incorporation and under-coordinated cations (such as Ag, Cu, Zn, and Sn) on the CZTSSe absorber surface [33], which leads to a shift in the binding energy. Besides, the intensity of the XPS peaks (including Ag, Cu, Zn, and Sn) demonstrates a significant decrease in the Ag/H co-doped CZTSSe sample, indicating that the hydrogen atoms released by the H-plasma treatment can passivate the surface defect states [42]. Additionally, a shoulder peak near the Sn 3*d* peak was observed in the CZTSSe-based samples, which corresponds to the Zn-Auger peak L3M45M45 [43]. It is clear that the Zn-Auger peak of the Ag/H co-doped sample is significantly weaker than that of the Reference and Ag-doped samples, implying that the Ag/H co-doping strategy can effectively inhibit the zinc-related defects in the CZTSSe-based samples [44], ultimately improving the performance of the final photovoltaic devices. Furthermore, the O 1*s* XPS spectrum can be fitted into two different peaks situated at 531.1 and 532.1 eV using a Gaussian fitting method, which correspond to the oxygen vacancies (V_O_) and O–H bonds (hydroxyl group) [45, 46], respectively. The O_1S_A: (O_1S_ A + O_1S_ B) ratio decreases from 0.70 to 0.62 after H-plasma treatment, indicating that the hydrogen incorporation can reduce the concentration of V_O_ defects [47]. The concentration of V_O_ defect for the three samples were further investigated by the electron paramagnetic resonance (EPR) spectra, as shown in Fig. S1c. It can be observed that the signal intensity of V_O_ (g = 2.003) gradually decreases along with the Ag and H doping, hinting a decrease in V_O_ defect concentrations [48], which is exactly in line with the XPS results. In addition, the increase in the intensity of O–H bonds in the Ag/H co-doped sample can well explain the enhancement of electrical and optical properties of CZTSSe thin films after Ag/H co-doping, as discussed below.

The effect of Ag-doping and Ag/H co-doping on the phase structure and morphology of the CZTSSe thin films were investigated using SEM, XRD, and Raman measurements. As shown in Fig. S2, the three samples exhibit a relatively uniform and dense morphology, signifying a good film quality. Further, the surface grain size significantly increased after Ag incorporation, which is in excellent agreement with literature reports on similar Ag-doped CZTSSe thin films [43]. Figure S3a illustrate the XRD patterns of the Reference, Ag-doped, and Ag/H co-doped samples. Three main diffraction peaks located at 27.31°, 45.26°, and 53.70° corresponding to the (112), (204)/(220), and (312) planes of tetragonal kesterite CZTSSe phase [2], respectively. Additionally, the other peaks, 2θ = 40.57°, were allocated to the Mo substrate [12]. The enlarged (112) diffraction peak of the Ag-doped and Ag/H co-doped CZTSSe samples tends to shift toward a lower angle compared with that of the reference sample, as presented in Fig. S3b. This result can be attributed to the replacement of smaller ionic radius Cu (0.74 Å) with larger Ag (1.14 Å) [35], thereby leading to the expansion of the unit cell volume. Besides, the full width half maximum (FWHM) of the Reference, Ag-doped, and Ag/H co-doped CZTSSe samples are determined by fitting the (112) peak of the XRD curves, as presented in Fig. S3c. It can be observed that the calculated FWHM for the Ag/H co-doped (0.1536) and Ag-doped (0.1558) samples are narrower than that of the Reference sample (0.1667), indicating that the crystallinity of the CZTSSe samples is significantly improved after Ag and H incorporation, which is the same as in Fig. S2. Further, the lattice constants a and c of the CZTSSe samples were derived from the XRD pattern (using JADE software) according to the literature method [49], as depicted in Tables S1-S3 (five samples for each group), and Fig. S3d. It is evident that the lattice constants a and c of the Ag/H co-doped sample (*a* = 5.674 Å, *c* = 11.346 Å) are slightly larger than those of the Ag-doped sample (*a* = 5.671 Å, *c* = 11.344 Å) and the reference sample (*a* = 5.660 Å, *c* = 11.320 Å), demonstrating that the existence of H ions in the CZTSSe lattices. The phase purity of the three samples were further investigated by Raman scattering, as shown in Fig. S3e. It is obvious that the Raman peaks located at 173, 196, and 240 cm^−1^ are assigned to the vibrational modes of kesterite CZTSe phase, and the Raman peaks located at 330 cm^−1^ is close to the vibrational mode of kesterite CZTS phase [16]. Furthermore, as exhibited in Fig. S3f, the Raman peaks of the CZTSSe samples near 196 cm^−1^ slightly shift toward lower-angle side after Ag and H incorporation, which is in good agreement with the XRD results. Apart from those, no other secondary phases characteristic peaks are detected in the three samples.

### Effect of Ag/H co-Doping on Photoelectric Properties Modulation

The electrical properties of the Reference, Ag-doped and Ag/H co-doped CZTSSe samples were investigated using Hall measurement, from which the hole carrier density (N_Hall_), resistivity, and Hall mobility can be acquired, the results are displayed in Fig. S4 and Table 1. The positive Hall coefficients indicate that all samples are P-type semiconductors [50, 51]. Compared with the Reference sample (0.63 cm^2^ V^−1^ s^−1^) and the Ag-doped sample (0.83 cm^2^ V^−1^ s^−1^), the Ag/H co-doped film (1.03 cm^2^ V^−1^ s^−1^) exhibits a relatively large Hall mobility, which may be attributed to the decreased cation disorder and the enhanced crystallinity induced by Ag and H doping [52, 53]. As anticipated, the resistivity of the CZTSSe samples increased from 2.48 × 10^2^ Ω cm (Reference sample) to a peak value of 7.15 × 10^2^ Ω cm (Ag-doped sample) and then decreased to 1.57 × 10^2^ Ω cm (Ag/H co-doped sample). On the contrary, the N_Hall_ of the three CZTSSe samples witness an opposite variation tendency to the resistivity, that is, the N_Hall_ values are 1.69 × 10^16^, 3.53 × 10^15^, and 3.85 × 10^16^ cm^−3^ for the Reference sample, Ag-based sample, and Ag/H-based sample, respectively. These results explicitly indicate that H doping in CZTSSe material is more beneficial to improve the poor electrical conductivity and the low hole carrier density caused by Ag substitution, which is in agreement with our expectation.

**Table 1:** Electrical properties of the Reference, Ag-doped, and Ag/H co-doped CZTSSe samples obtained from the Hall measurement

Sample	Hole carrier density (cm^−3^)	Resistivity (Ω cm)	Hall mobility (cm^2^ V^−1^ s^−1^)
Reference	1.69 × 10^16^	2.48 × 10^2^	0.63
Ag-doped	3.53 × 10^15^	7.15 × 10^2^	0.83
Ag/H co-doped	3.85 × 10^16^	1.57 × 10^2^	1.03

**Fig. 3:**
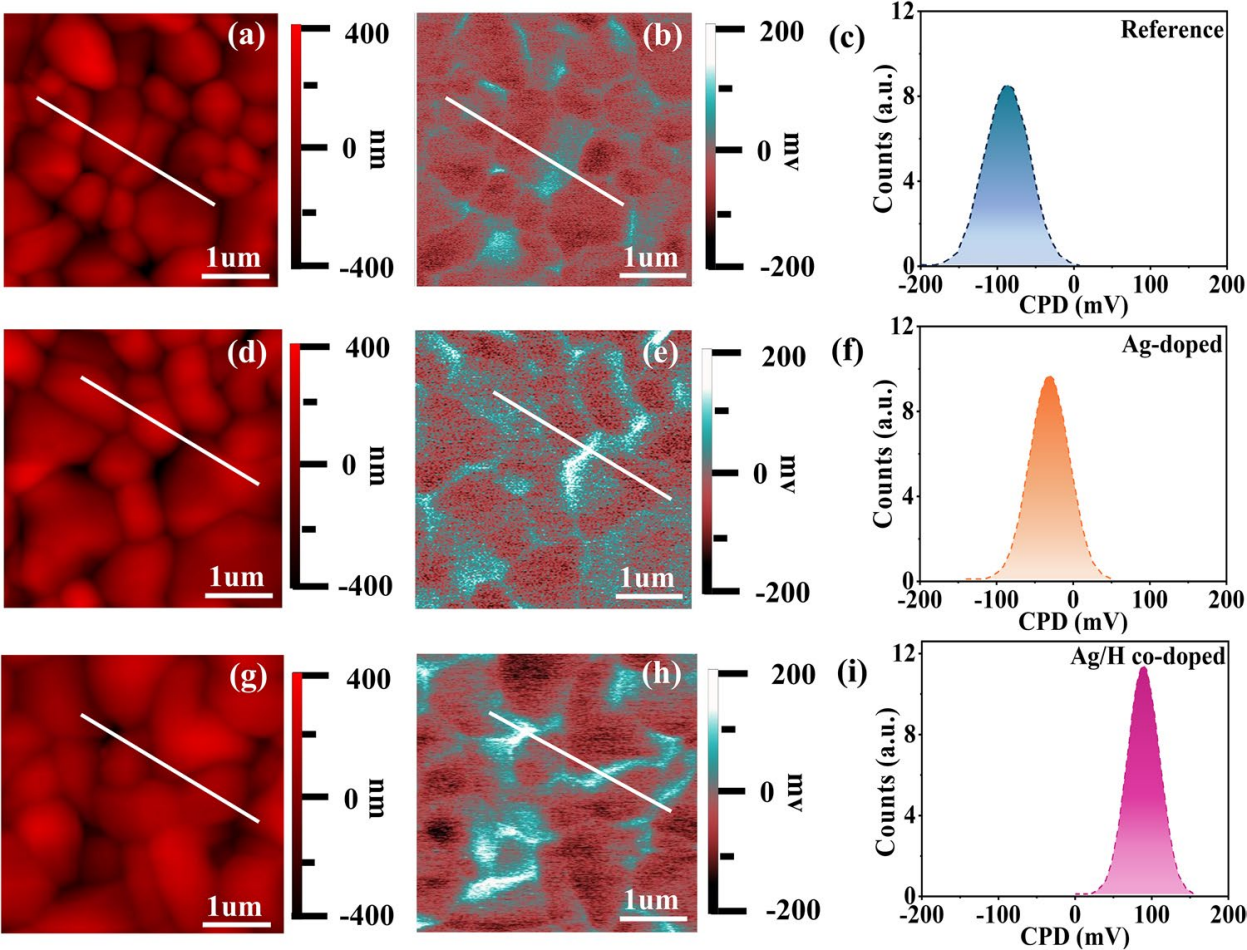
KPFM measurement on CZTSSe thin films: **a**–**c** Reference, **d–f** Ag-doped, and **g–i** Ag/H co-doped samples. **a**, **d**, and **g** AFM topography images. **b**, **e** and **h** KPFM surface potential images. **c**, **f** and **i** Surface contact potential difference (CPD) distribution. The scan size is 4 × 4 μm^2^

Kelvin probe force microscopy (KPFM) was performed to investigate the surface potential distribution of the CZTSSe thin films before and after treatment with Ag/H. As depicted in Fig. 3a, d, and g, the grain size of the CZTSSe sample is significantly increased after Ag and H treatment, which is the same as the SEM results (Fig. S2). From Figs. 3b, e, h, and S5a-c, it can be observed that the surface potential of the grain boundaries (GBs) in the three samples are higher than that of the intragrains (IGs). This result demonstrates a positive surface potential at the GBs and a negative potential in the IGs, thereby leading to downward band-bending at the GBs (Fig. S5d–f), which results in reduced carrier recombination and enhanced carrier transport. The distribution histogram of the surface contact potential difference (CPD) for the three samples are shown in Fig. 3c, f, and i. It can be observed that the Ag/H co-doped sample exhibits a narrower CPD distribution compared to the Reference sample and Ag-doped sample, suggesting that Ag/H co-doping is more beneficial to improve the uniformity of electrical properties. Besides, the average contact potential difference (CPD) of the three samples are − 87 mV (Reference), − 29 mV (Ag-doped), and 85 mV (Ag/H co-doped), respectively. As one knows, a higher CPD means a higher Fermi level [5, 54]. Obviously, the Ag/H co-doped CZTSSe thin film exhibits a higher E_F_, which is beneficial to increase the formation energy of Sn_Zn_ defects [29], thereby inhibiting the Sn_Zn_-related defects.

### Photovoltaic Performance of the CZTSSe Solar Cells

The photovoltaic devices were then fabricated with a structure of soda-lime glass/Mo/CZTSSe/CdS/i-ZnO/ITO/Ag. The concentration of Ag was first optimized. All the CZTSSe solar cells were fabricated from the same batch to provide analytical consistency. The performances of the solar cells were measured under AM 1.5 G illumination with an active area of 0.21 cm^2^. Figures S6a and S8 display the current density–voltage (*J–V*) curves and the statistical boxplots of the CZTSSe solar cells with the concentration of Ag varying from 0 to 20 wt%. The detailed device parameters are reported in Tables S5 and S6. It can be observed that the CZTSSe device with 15 wt% Ag substitution exhibits an excellent PCE (12.54%), which may be attributed to the enhanced crystallinity and the decreased defect densities, as mentioned in a previous publication [18]. However, the performance of the CZTSSe solar cells decreases gradually instead of increasing when the Ag content is further increased to 20 wt%, which may be explained by the poor electrical conductivity and the low carrier density for the CZTSSe material at a higher Ag doping levels usually leads to a dramatic decrease in carrier transport process [39].

The hydrogen concentration was then optimized based on the concentration of Ag is 15 wt%. Figures S6b and S9 present the *J-V* curves and the boxplots for six series of Ag-based CZTSSe devices with the concentrations of H_2_ varying from 0 to 10%. The statistical distributions of the photovoltaic parameters are summarized in Tables S7 and S8. It can be observed that the PCE of Ag/H co-doped device increases dramatically and reaches a peak at the concentration of H_2_ is 6% and then decreases when the hydrogen concentration is further increased to 8% and 10%. The champion device exhibits a *V*_OC_ of 535.37 mV, FF of 68.20%, and *J*_SC_ of 36.97 mA cm^−2^, leading to a PCE of 13.50% (Table S7). To further optimize the device performance, the effect of H-plasma treatment power and H-plasma treatment time on the final devices were successively investigated based on a concentration of H_2_ of 6%. Figures S10 and S6c show the statistical distributions of the photovoltaic parameters and *J-V* curves for the photovoltaic devices with the H-plasma treatment powers varying from 0 to 80 W, the detailed results are summarized in Tables S9 and S10. It can be observed that the best performance is obtained when the H-plasma treatment power is 50 W. However, when the treatment power is further increased to 60 and 80 W, the device performance decreases gradually instead of increasing, probably due to the elevated ionization rate of H_2_ at high power [55]. The champion device exhibits a PCE of 14.22%, with a *V*_OC_ of 555.92 mV, an FF of 68.54%, and a *J*_SC_ of 37.32 mA cm^−2^ (Table S9). Figures S6d and S11 present the *J-V* curves and the corresponding statistical distributions of the photovoltaic parameters for CAZTSSe devices with the H-plasma treatment times varied in the range of 0–40 s, the detailed results are listed in Tables S11 and S12. It can be observed that the efficiency of the CAZTSSe device is significantly improved with a gradually increased H-plasma treatment time until its limited (25 s) is reached. The best device delivers an improved PCE of 14.57%, with a *V*_OC_ of 562.45 mV, an FF of 69.42%, and a *J*_SC_ of 37.33 mA cm^−2^ (Table S11). Based on these results, the CAZTSSe absorber received a H-plasma treatment procedure with the H_2_ concentration of 6%, the H-plasma treatment power of 50 W, and the H-plasma treatment time of 25 s delivers the champion efficiency.

**Fig. 4:**
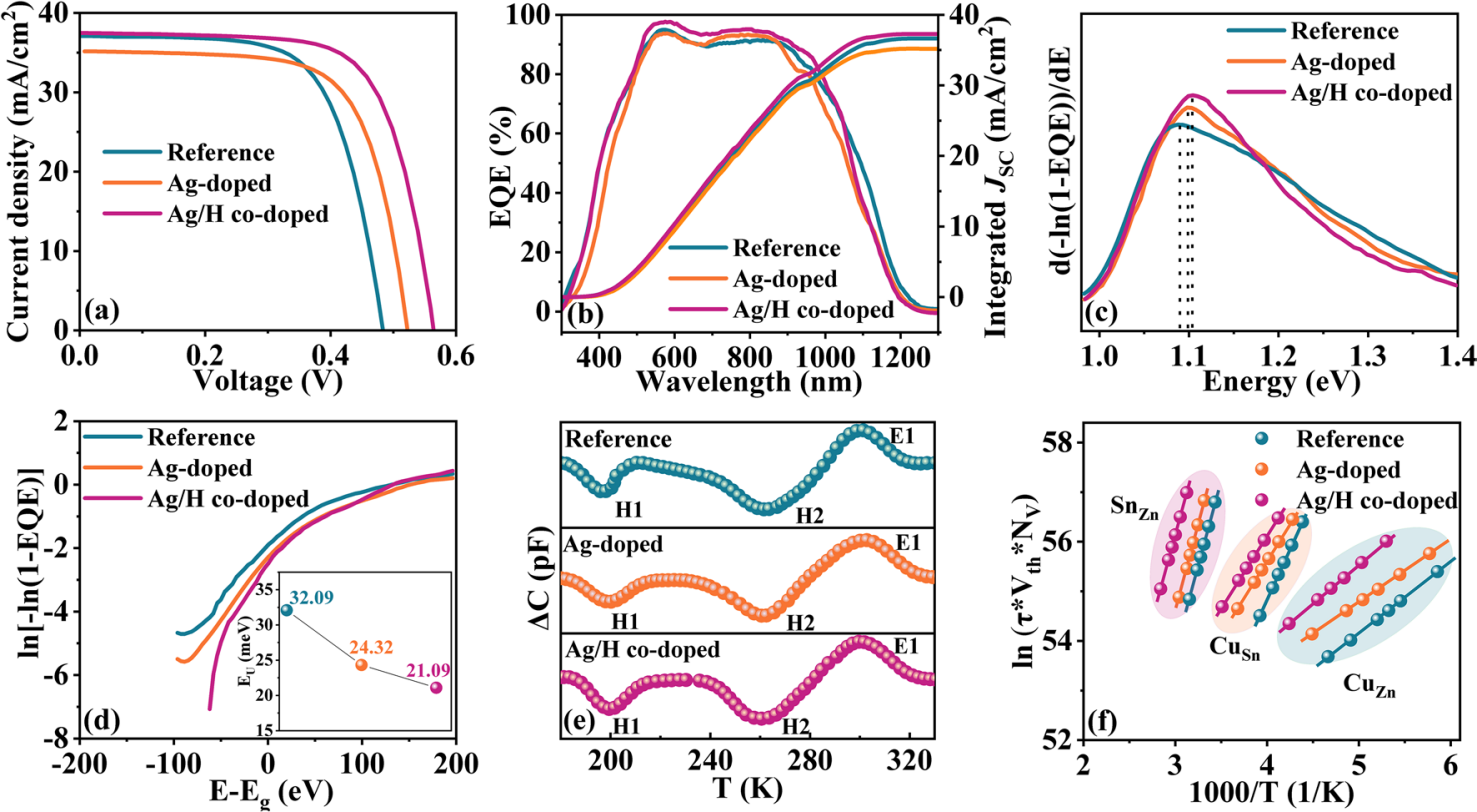
**a** *J–V* curves. **b** EQE spectra of the CZTSSe solar cells and the integrated *J*_SC_. **c** Bandgaps derived from the EQE curves. **d** Urbach tail energy (E_U_) extracted from the EQE data. **e** Deep-level transient spectroscopy (DLTS) signals of the CZTSSe devices, and **f** the corresponding Arrhenius plots

The representative *J–V* curves of the Reference, Ag-doped, and Ag/H co-doped CZTSSe devices are shown in Fig. 4a and the details of device parameters are listed in Table 2. The corresponding statistical distributions of the photovoltaic parameters are also provided in Table S4 and Fig. S7. It is clearly visible that the three series of photovoltaic devices exhibit a convincing reproducibility and reliability. Benefiting from the synergistic optimization effects brought by Ag substitution and H incorporation, the Ag/H co-doped CZTSSe device yields an exceptional PCE of 14.74%, with a *V*_OC_ of 564.07 mV, an FF of 69.71%, and a *J*_SC_ of 37.49 mA cm^−2^. In contrast, the reference device offers an inferior PCE of 11.94%, with a *V*_OC_ of 483.32 mV, an FF of 66.59%, and a *J*_SC_ of 37.10 mA cm^−2^. The improvement in PCE for the Ag/H co-doped device mainly comes from the enhanced *V*_OC_, which may be mainly attributed to the reduction of defect-related non-radiative recombination, as discussed below.

**Table 2:** Detailed photovoltaic parameters of the Reference, Ag-doped, and Ag/H co-doped CZTSSe devices

Sample	PCE (%)	*V*_OC_ (mV)	*J*_SC_ (mA cm^2^)	FF (%)	E_g_ (eV)	E_U_ (meV)	R_S_ (Ω cm^2^)	R_SH_ (Ω cm^2^)
Reference	11.94	483.32	37.10	66.59	1.089	32.09	0.50	882.28
Ag-doped	12.62	522.55	35.17	68.67	1.100	24.32	0.76	1395.05
Ag/H co-doped	14.74	564.07	37.49	69.71	1.102	21.09	0.45	1821.53

To elucidate the role of Ag/H co-doping on the device performance, the series resistance (*R*_S_) and shunt resistance (*R*_SH_) of the CZTSSe solar cells were determined by fitting the light *J − V* curves according to the method reported in the literature [15, 56], as presented in Fig. S12 and Table 2. In principle, FF is directly proportional to *R*_SH_ and inversely proportional to *R*_S_, while *V*_OC_ is directly proportional to *R*_SH_ [39]. Obviously, the Ag/H co-doped device demonstrates a small *R*_S_ and a large *R*_SH_ compared to the reference and Ag-doped samples, indicating that the Ag/H co-doping in CZTSSe sample is favorable for improving carrier transport and collection, which consequently enhances the FF and *V*_OC_ of the resulting CZTSSe devices. Besides, for the Ag-doped CZTSSe device, the increased *R*_S_ can be ascribed to the increased resistivity, while the increased *R*_SH_ is at least partially attributable to the improved crystallinity, as demonstrated in the previous section.

Figure 4b presents the external quantum efficiency (EQE) spectra and the corresponding integrated current of the CZTSSe devices. Notably, the Ag/H co-doped device exhibits a stronger EQE response than the Reference and Ag-doped devices in the wavelength range of 500–1000 nm, demonstrating a reduction of recombination losses of photogenerated carriers in the Ag/H co-doped device. The integrated *J*_SC_ values are 36.63, 35.16, and 37.26 mA cm^−2^ for the Reference, Ag-doped, and Ag/H co-doped CZTSSe solar cells, respectively, which confirmed the reliability of *J*_SC_ in the previous *J-V* measurement (Fig. 4a). As exhibited in Fig. 4c and Table 2, the calculated E_g_ values from the EQE plots for the CZTSSe solar cells is slightly increased from 1.089 eV (Reference device) to 1.100 eV (Ag-doped device) and 1.102 eV (Ag/H co-doped device), which is in line with the previous publication [57]. Besides, the Urbach energy (E_U_) is extracted for the three photovoltaic devices (Fig. 4d), reading 32.09 meV for the Reference device, 24.32 meV for the Ag-doped device, and 21.09 meV for the Ag/H co-doped device (Table 2). As is well-known, a lower E_U_ value indicates a reduction in energetic disorder at the band edge of the photovoltaic device [43, 58]. After Ag/H co-doping, the optimized E_U_ value is more beneficial to reduce energy loss and facilitating carrier transport, corresponding directly to higher PCE in the final devices.

### Effect of Ag/H co-Doping on Defect Passivation

Capacitance-mode deep-level transient spectroscopy (C-DLTS) measurement was performed on soda-lime glass/Mo/CZTSSe/CdS/i-ZnO/ITO/Ag-grid structure to further explore the origin of the enhancement of cell performance. The measurement was carried out in the temperature range of 180 to 340 K, as shown in Fig. 4e. Three peaks are clearly observed in C-DLTS curves for the three samples, implying that three defects appeared in the test range. Subsequently, the corresponding activation energy (E_a_) and defect density (N_T_) of the detected defect are derived from C-DLTS spectra based on the method described in detail elsewhere [39], the results are presented in Fig. 4f and Table 3. It is reported that the *E*_a_ of 0.10–0.18, 0.25–0.38, and 0.55–0.62 eV corresponded well to Cu_Zn_, Cu_Sn_, and Sn_Zn_ defects, respectively [12, 24, 34, 59]. It is well known that these three kinds of defects are all deep level defects in CZTSSe cells [17, 19, 24, 29, 59], which are detrimental to the performance of the final devices. Notably, for these three types of defects, the defect activation energies of the Ag/H co-doped device are smaller than those of the reference device and the Ag-doped device. Additionally, for the Cu_Zn_ and Cu_Sn_ antisite defects, the Ag/H co-doped sample demonstrates lower defect densities than the reference and Ag-doped samples. More importantly, as anticipated above, the density of Sn_Zn_ donor defects was deduced to be 1.17 × 10^13^ cm^−3^ for the Ag/H co-doped device, which is much lower than that of the reference device (8.01 × 10^14^ cm^−3^) and the Ag-doped sample (1.07 × 10^14^ cm^−3^). The reduced defects density of Sn_Zn_ donor is mainly ascribed to the C=O and O–H functional groups that induced by hydrogen incorporation, can interact with under-coordinated cations in CZTSSe absorber, thereby enabling the Sn_Zn_-related defects to be suppressed.

**Table 3:** Summary of the relevant parameters of the observed defect levels for the Reference, Ag-doped, and Ag/H co-doped CZTSSe devices

Sample	Peak ID	E_a_ (eV)	N_T_ (cm^−3^)	Possible defect level
Reference	H1	0.165	5.70 × 10^15^	Cu_Zn_
	H2	0.353	3.54 × 10^14^	Cu_Sn_
	E1	0.615	8.01 × 10^14^	Sn_Zn_
Ag-doped	H1	0.135	4.63 × 10^14^	Cu_Zn_
	H2	0.261	7.31 × 10^13^	Cu_Sn_
	E1	0.607	1.07 × 10^14^	Sn_Zn_
Ag/H co-doped	H1	0.103	1.92 × 10^14^	Cu_Zn_
	H2	0.253	3.98 × 10^13^	Cu_Sn_
	E1	0.578	1.17 × 10^13^	Sn_Zn_

**Fig. 5:**
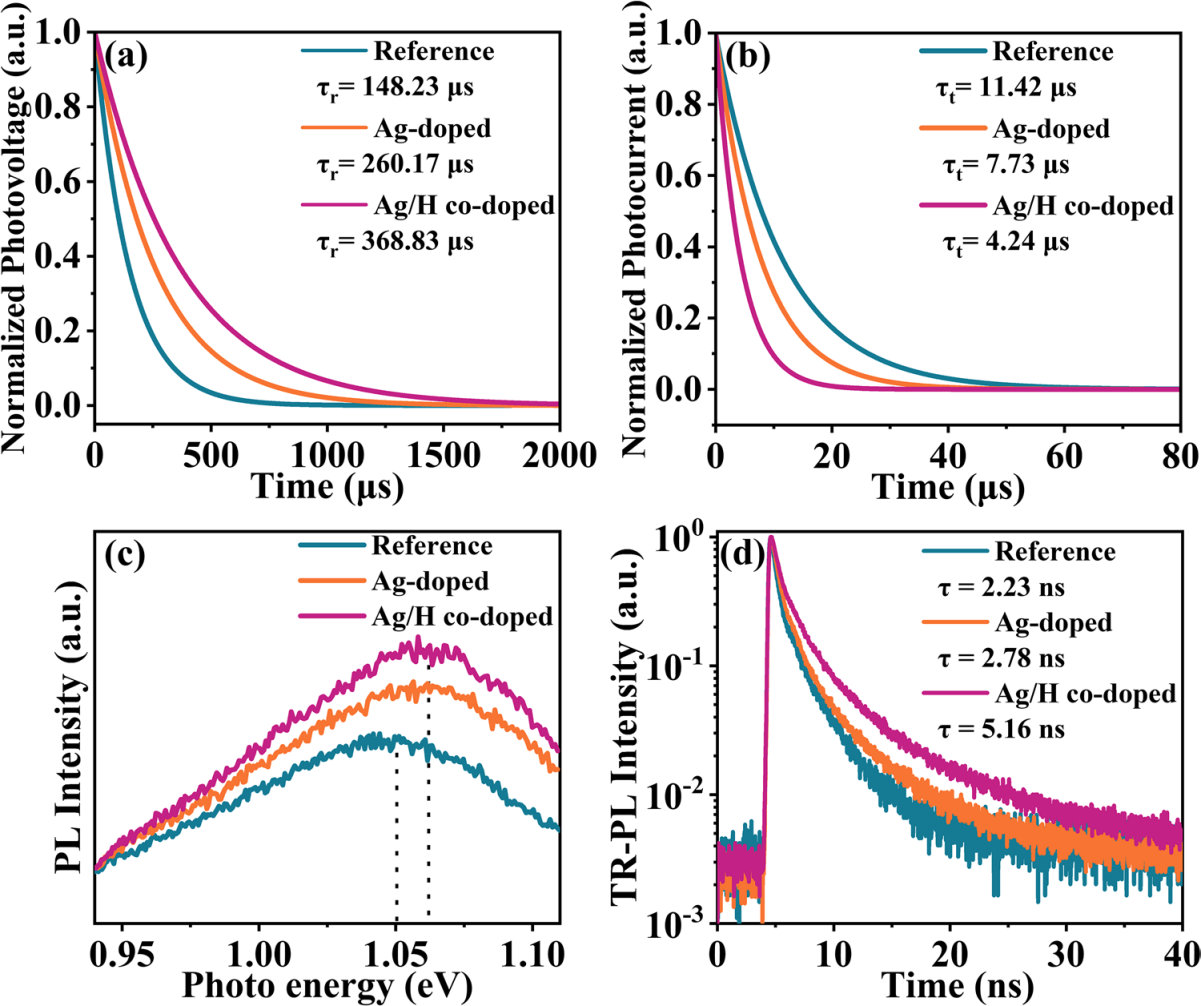
**a** Normalized transient photovoltage (TPV), and **b** transient photocurrent (TPC) spectra of the Reference, Ag-doped, and Ag/H co-doped CZTSSe solar cells. **c** Steady-state photoluminescence (PL) spectra, and **d** time-resolved TRPL curves of the CZTSSe devices

Subsequently, electrochemical impedance spectroscopy (EIS), transient photovoltage (TPV), and transient photocurrent (TPC) measurements were performed to understand the carrier transfer and recombination characteristics of the CZTSSe solar cells. The Nyquist plots of the CZTSSe devices are depicted in Fig. S13a. It is clear that the Ag/H co-doped device exhibits a small series resistance (*R*_O_) and a large recombination resistance (*R*_ct_) compared to the Reference and Ag-doped samples, manifesting that the Ag/H co-doping in CZTSSe device is beneficial to the enhancement of carrier transport and suppressing carrier recombination, which eventually elevates the resulting cell performance. The carrier transfer features of the CZTSSe solar cells were further investigated with TPV/TPC measurement. As depicted in Fig. 5a, the charge recombination lifetime (τ_r_) significantly increases from 148.23 µs (Reference sample) to 260.17 µs (Ag-doped sample) and 368.83 µs (Ag/H co-doped sample), indicating that the carrier non-radiative losses are effectively inhibited within the Ag/H co-doped sample. As illustrated in Fig. 5b, the charge-transfer lifetime (τ_t_) are about 11.42, 7.73, and 4.24 µs for the Reference, Ag-doped, and Ag/H co-doped devices, respectively. This result indicates that the charge extraction and transport is relatively fast in the Ag/H co-doped CZTSSe device. In addition, as shown in Fig. 5c, the photoluminescence (PL) emission peak energy (E_PL_) of the Reference, Ag-doped, and Ag/H co-doped devices are 1.050, 1.060, and 1.061 eV, respectively. The increased PL intensity and improved E_PL_ for the Ag/H co-doped CZTSSe sample indicate that the deep-level defect and trap-assisted non-radiative recombination in the CZTSSe samples are significantly suppressed. Furthermore, the carrier transfer features were further scrutinized via the time-resolved photoluminescence (TRPL) measurement. As shown in Fig. 5d, the minority carrier lifetime (τ) is significantly increased from 2.23 to 2.78 and 5.16 ns for the Reference, Ag-doped, and Ag/H co-doped CZTSSe samples, respectively. The prolongation on τ clearly indicates that the undesired non-radiative recombination in the CZTSSe absorber is effectively suppressed after Ag/H co-doping, closely related to the improved device efficiency. All these results can well explain the excellent cell performance achieved in Ag/H co-doped CZTSSe solar cells.

**Fig. 6:**
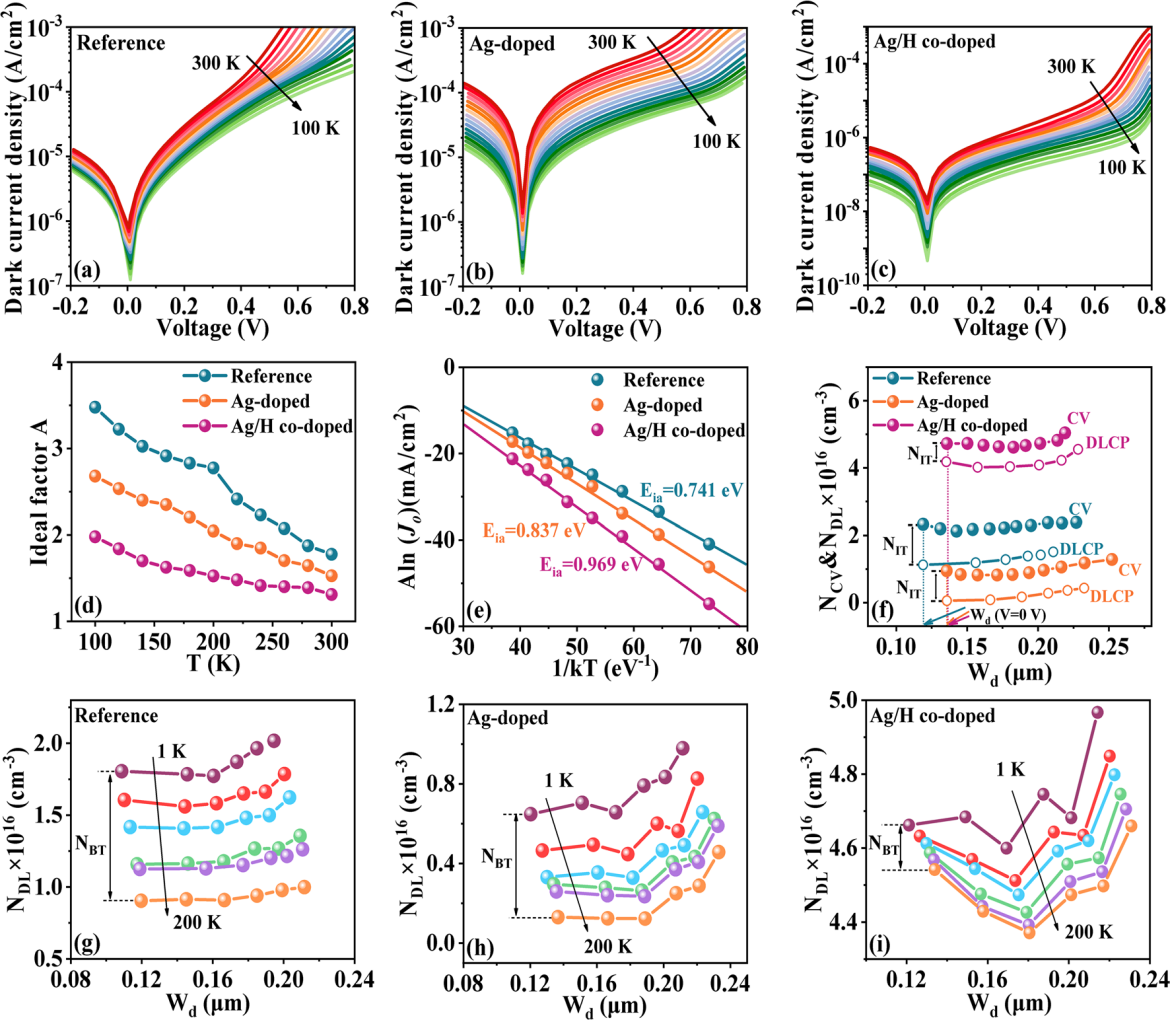
Temperature-dependent dark *J-V* curves: **a** Reference, **b** Ag-doped, and **c** Ag/H co-doped CZTSSe samples. **d** Ideal factor A. **e** Temperature correlation Aln (J_0_) versus 1/kT curves of the Reference, Ag-doped and Ag/H co-doped CZTSSe devices. **f** C-V and DLCP profiles of the CZTSSe devices. N_DL_ of the **g** Reference, **h** Ag-doped, and **i** Ag/H co-doped devices measured at frequencies from 1 to 200 kHz

To further elucidate the dominant recombination mechanism of the CZTSSe photovoltaic devices, temperature-dependent dark *J–V* (*J–V–T*) measurement was carried out. Figure 6a-c displays the *J-V-T* curves for the Reference, Ag-doped, and Ag/H co-doped CZTSSe devices, respectively, performed in a temperature range of 100–300 K under dark condition. Notably, the diode behaviour of the Ag/H co-doped device is significantly stronger than that of the Reference and Ag-doped samples in the test range, emphasizing that the Ag/H co-doping strategy have a significant effect on the charge collection efficiency. Moreover, compared with the Reference sample (Fig. 6d), the Ag-doped sample shows a small diode ideality factor (A) and a low temperature dependence. In addition, the most significant reduction in the diode ideality factor and temperature dependence is found in the Ag/H co-doped device, hinting that the carrier recombination losses during the carrier transport process are effectively suppressed. The recombination activation energies (*E*_ia_) of the solar cells can be extracted from the dark *J-V-T* curves based on the methods described in detail elsewhere [17]. As indicated in Fig. 6e, the E_ia_ values of the Reference, Ag-doped, and Ag/H co-doped CZTSSe devices are, respectively, 0.741, 0.837, and 0.969 eV. As is well-known, the value of *E*_g_-*E*_ia_ (E_g_, extracted from the EQE data) provides the information of the dominant recombination path, that is, interface recombination or Shockley-Reed-Hall (SRH) recombination. It is apparently that the Ag/H co-doped CZTSSe device has a comparatively large *E*_ia_ of 0.969 eV, relatively close to its *E*_g_ of 1.102 eV. This result hints that Ag/H co-doping strategy can effectively inhibit the interface recombination process, ultimately giving rise to higher *V*_OC_ and better cell performance.

The capacitance–voltage (*C–V*) and drive level capacitance profiling (DLCP) measurements were performed to profoundly investigate the carrier density and the defect properties of the CZTSSe solar cells, the results are presented in Fig. 6f and Table S13. It can be observed that the depletion width (*W*_d_) of the Ag-doped and Ag/H co-doped devices are slightly larger than that of the Reference sample, which is more beneficial to photo-generated carrier collection in the photovoltaic devices. In addition, the built-in voltage (*V*_bi_) of the CZTSSe samples can be determined from the *C-V* curves by mapping 1/*C*^2^ *vs. V*, as presented in Fig. S13b. Apparently, the built-in voltage (*V*_bi_) increases from 0.626 V (Reference device) to 0.748 V (Ag-doped device) and 0.879 V (Ag/H co-doped device), hinting a stronger driving force for photon-generated carrier collection and separation in Ag/H co-doped devices, which correlate well with the EIS, TPV and TPC results. Additionally, the interface defect density (N_IT_) can be identified by the difference between *C-V* (N_C–V_) and DLCP (N_DLCP_) at zero bias, following the methods described in detail by Liang et al. [58]. Obviously, the N_IT_ for the CZTSSe devices is decreased from 1.20 × 10^16^ cm^−3^ (Reference device) to 8.89 × 10^15^ cm^−3^ (Ag-doped device) and 5.40 × 10^15^ cm^−3^ (Ag/H co-doped device). This result further indicates that Ag-doping and Ag/H co-doping have a noticeable effect on the interface-recombination process of the photovoltaic devices [60], and Ag/H co-doping is more effective than Ag-doping. As shown in Fig. 6g–i, the bulk defect density (N_BT_) of the CZTSSe devices can be extracted from the difference between low-frequency and high-frequency DLCP. The values of N_BT_ is calculated as 9.01 × 10^15^, 5.17 × 10^15^, and 1.21 × 10^15^ cm^−3^ for the Reference, Ag-doped, and Ag/H co-doped devices, respectively, implying that Ag/H co-doping extremely facilitates the reduction of bulk defect density, ultimately improving the device performance. All these results explicitly indicate that the Ag/H co-doping strategy is highly conducive to the carrier extraction and transport in CZTSSe solar cells, ultimately delivering overall improved device performance.

## Conclusions

In conclusion, we have demonstrated a methodological approach for the effective passivation of Sn_Zn_-related defects utilizing Ag/H co-doping strategy to enhance the performance of CZTSSe devices. The H doping effectively enhanced the electrical conductivity as well as increased the carrier density of the Ag-based CZTSSe absorbers. The dramatical reduction of defect densities for Sn_Zn_ donor is attributed to electron-donating properties of the C=O and O–H functional groups induced by the H-plasma treatment, which interplays with the under-coordinated cations in CZTSSe materials. The in-depth investigation results reveal that the Ag/H co-doping strategy in CZTSSe photovoltaic devices can effectively reduce the detrimental band tailing, extend the minority carrier lifetime, and more important, suppress the carrier non-radiative recombination and enhance the carrier extraction and transport. Consequently, the Ag/H co-doped CZTSSe device exhibits an impressive PCE of 14.74%, with a *V*_OC_ of 564.07 mV, an FF of 69.71%, and a *J*_SC_ of 37.49 mA cm^−2^. These results offer deeper insights into the internal loss mechanism of CZTSSe materials, paving the way for future optimizations toward high-efficiency kesterite-based photovoltaic devices.

## Supplementary Information

Below is the link to the electronic supplementary material.

Supplementary file1 (DOCX 2002 KB)
