# Supplementary material for: Revealing the Role of Hydrogen in Highly Efficient Ag-Substituted CZTSSe Photovoltaic Devices: Photoelectric Properties Modulation and Defect Passivation
Source: Nanomicro Lett. 2024 Dec 3;17:84. doi: 10.1007/s40820-024-01574-3 (PMC11615170; doi:10.1007/s40820-024-01574-3)
Supplement: Supplementary file 1 — Supplementary file1 (DOCX 2002 KB) [file 40820_2024_1574_MOESM1_ESM.docx]

Supporting Information for

**Revealing the Role of Hydrogen in Highly Efficient Ag-Substituted CZTSSe Photovoltaic Devices: Photoelectric Properties Modulation and Defect Passivation**

Xiaoyue Zhao^1^, Jingru Li^1^, Chenyang Hu1, Yafang Qi^1^,*, Zhengji Zhou^1^, Dongxing Kou^1^, Wenhui Zhou^1^, Shengjie Yuan^1^, and Sixin Wu^1^,*

^1^The Key Laboratory for Special Functional Materials of MOE, School of Nanoscience and Materials Engineering, National & Local Joint Engineering Research Center for High-efficiency Display and Lighting Technology, Collaborative Innovation Center of Nano Functional Materials and Applications, Henan University, Kaifeng 475004, P. R. China

*Corresponding authors. E-mail: [qiyafang@henu.edu.cn](mailto:qiyafang@henu.edu.cn) (Yafang Qi); [wusixin@henu.edu.cn](mailto:wusixin@henu.edu.cn) (Sixin Wu)

**Supplementary Figures and Tables**


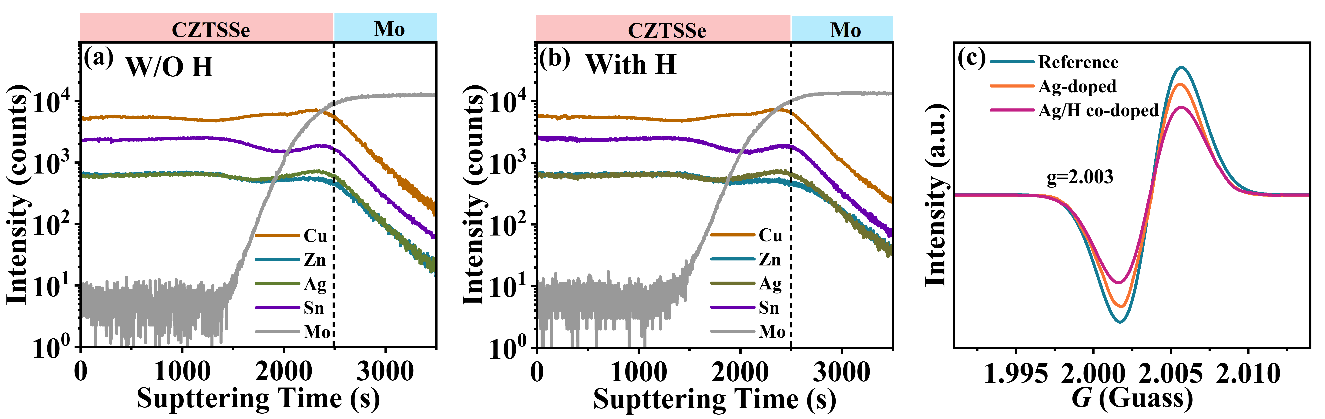


**Fig. S1** Element distribution TOF-SIMS depth profiles of the CAZTSSe thin films without (**a**) and with (**b**) H-doping. (**c**) The EPR spectra of the Reference, Ag-doped, and Ag/H co-doped CZTSSe absorbers


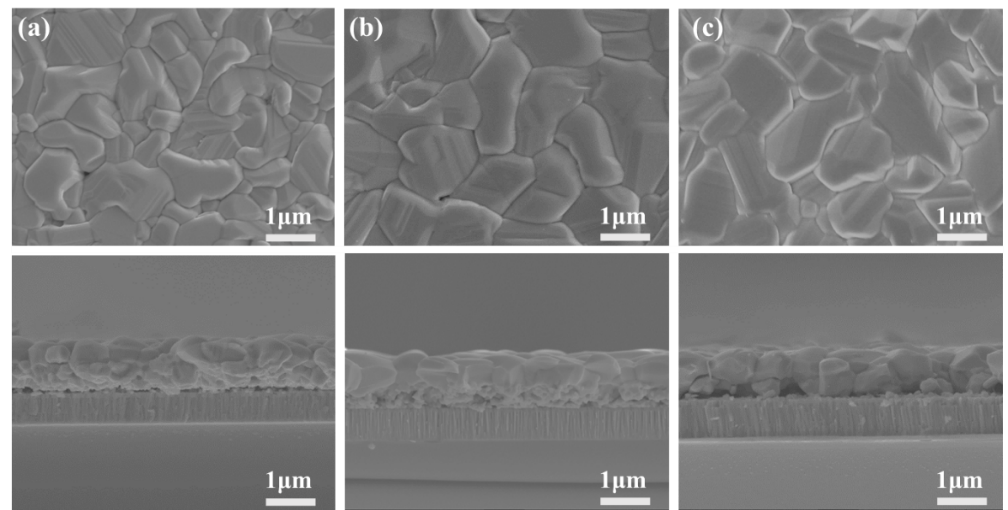


**Fig. S2** Top-view and cross-sectional SEM images of the CZTSSe thin films: **(a)** Reference, **(b)** Ag-doped, and **(c)** Ag/H co-doped samples


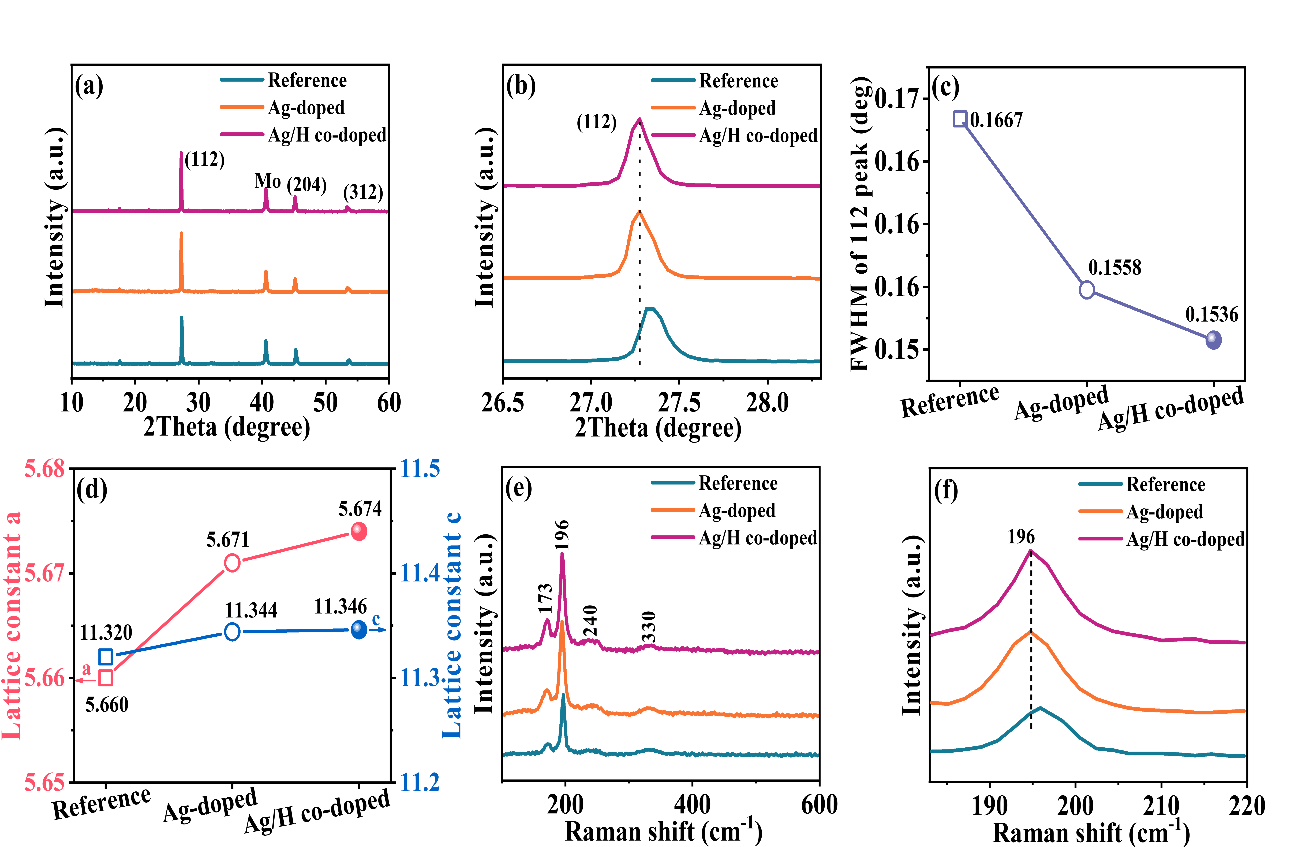


**Fig. S3 (a)** X-ray diffraction (XRD) patterns of CZTSSe samples, **(b)** the magnified (112) peaks, **(c)** The FWHM of (112) peaks. **(d)** The lattice constants a and c for the Reference, Ag-doped, and Ag/H co-doped absorbers, and **(e)** Raman spectra of CZTSSe samples. **(f)** The enlarged view of the Raman peak located at 196 cm^-1^


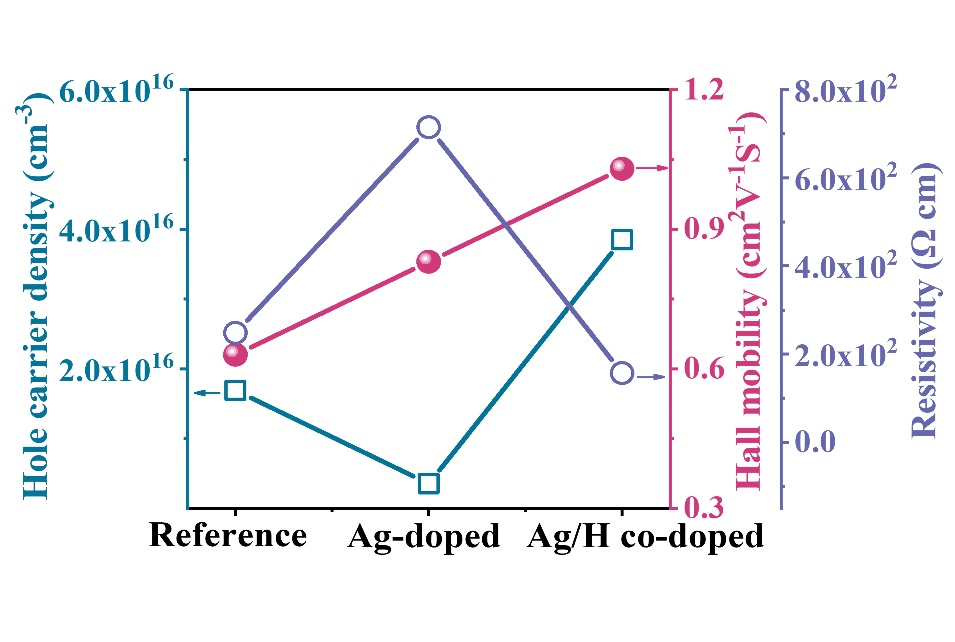


**Fig. S4** Hole carrier density, Hall mobility, and Resistivity of the Reference, Ag-doped, and Ag/H co-doped samples obtained by Hall measurement


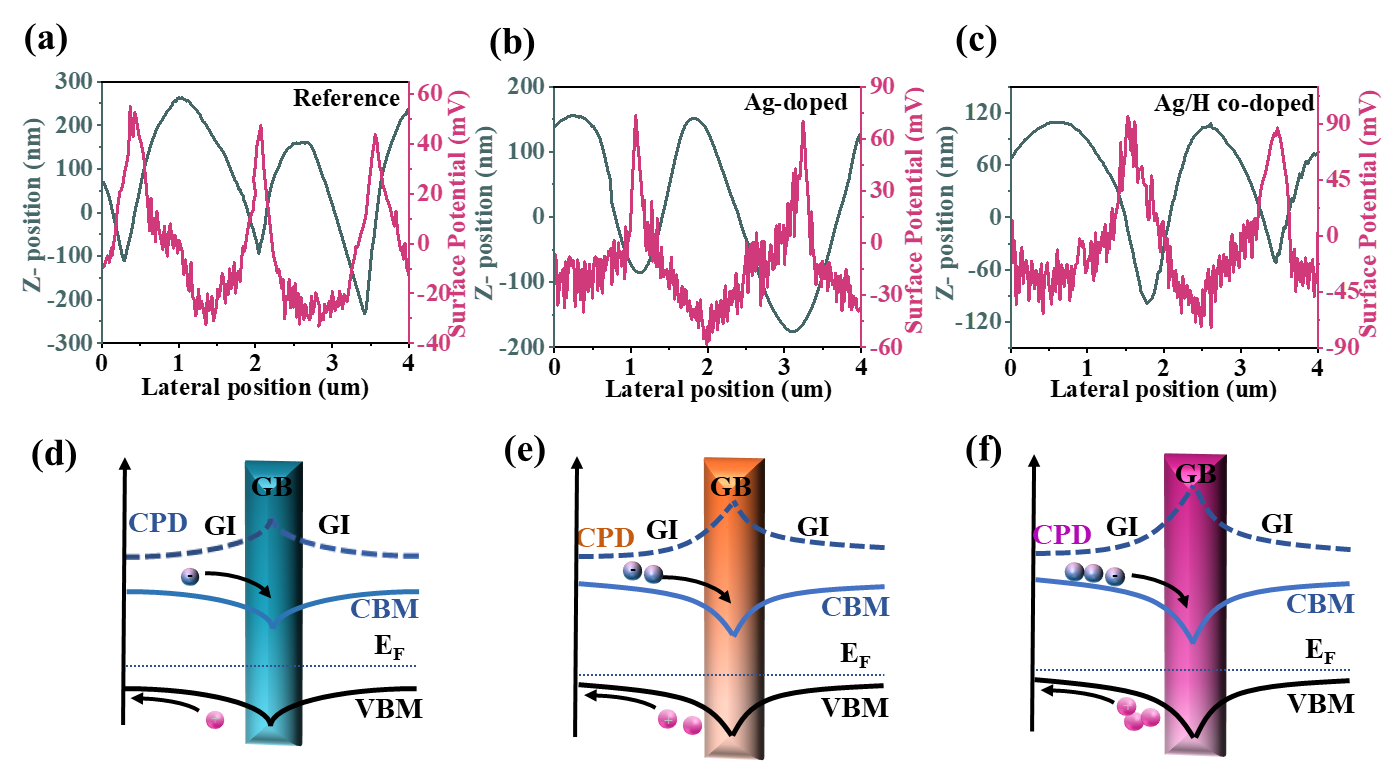


**Fig. S5** Plots of the surface topography and surface potential line scan for the **(a)** Reference film **(b)** Ag-doped film and **(c)** Ag/H co-doped film. The schematic diagrams of band bending near GBs for the CZTSSe absorbers: **(d)** Reference, **(e)** Ag-doped, and **(f)** Ag/H co-doped samples


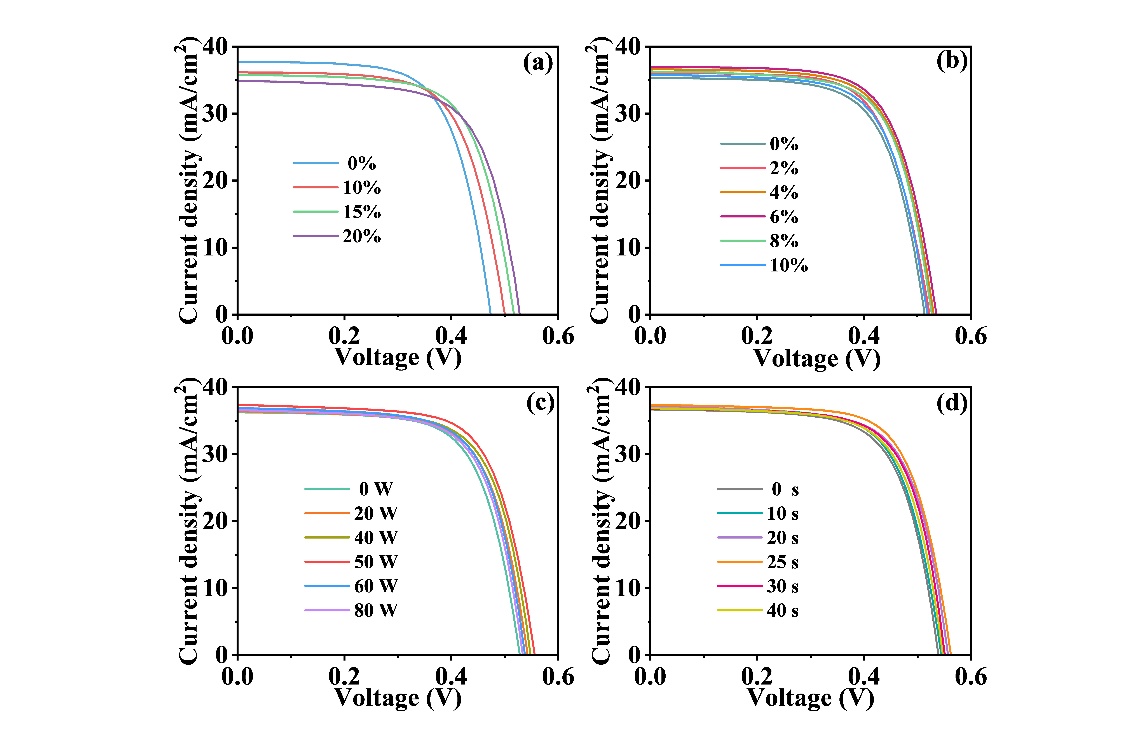


**Fig. S6** **(a)** The *J-V* curves of the champion device with different Ag concentrations (0 wt%, 10 wt%, 15 wt%, and 20 wt%). **(b)** *J-V* curves of the champion device with different H_2_ concentrations (0%, 2%, 4%, 6%, 8%, and 10%). **(c)** *J-V* curves of the champion device with different H-plasma treatment powers (0, 20, 40, 50, 60, and 80 w). **(d)** *J-V* curves of the champion device with different H-plasma treatment times (0, 10, 20, 25, 30, and 40 s).


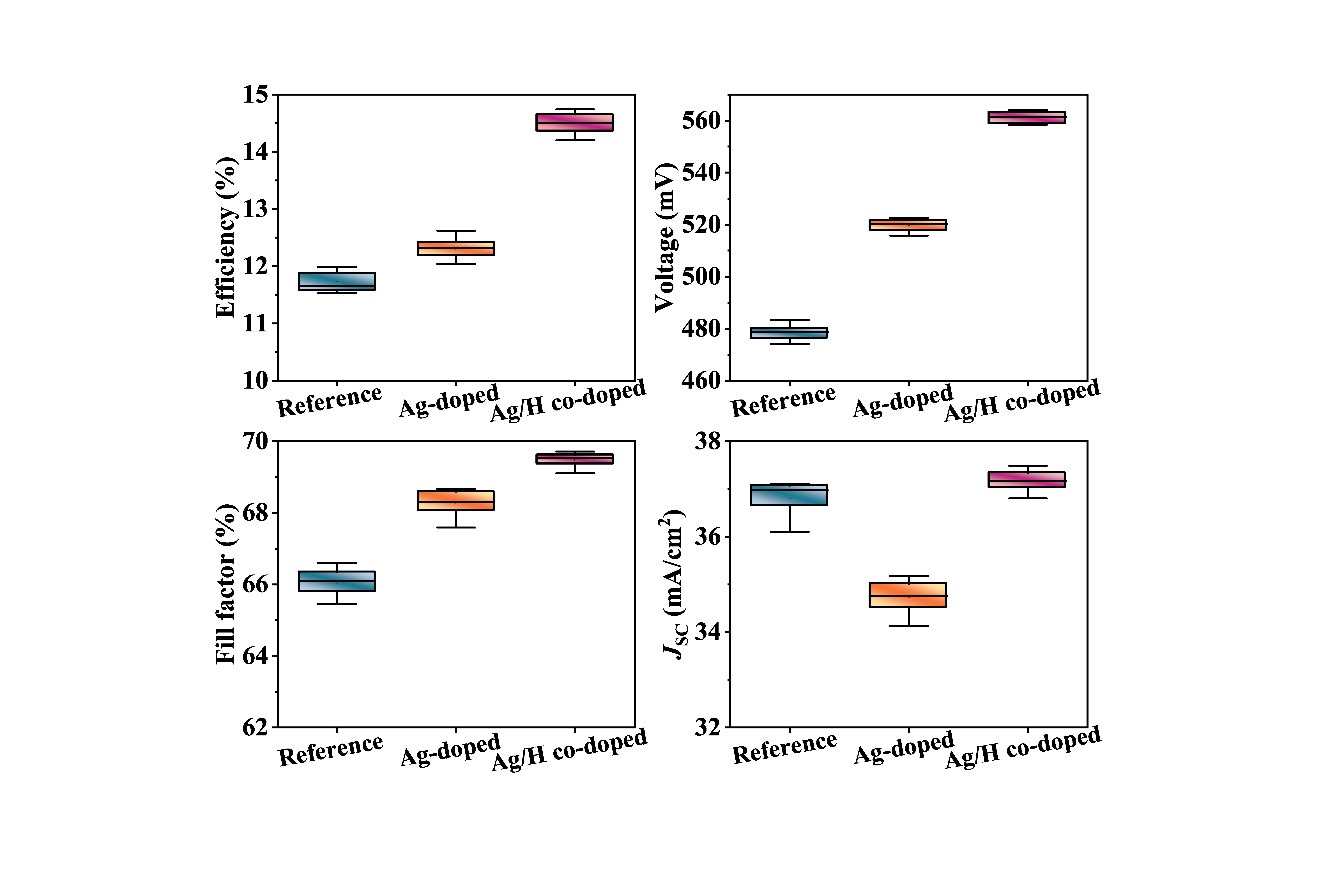


**Fig. S7** Statistical box plots of photovoltaic parameters for the Reference, Ag-doped, and Ag/H co-doped solar cells. Data are extrapolated from the analysis of 18 solar cells


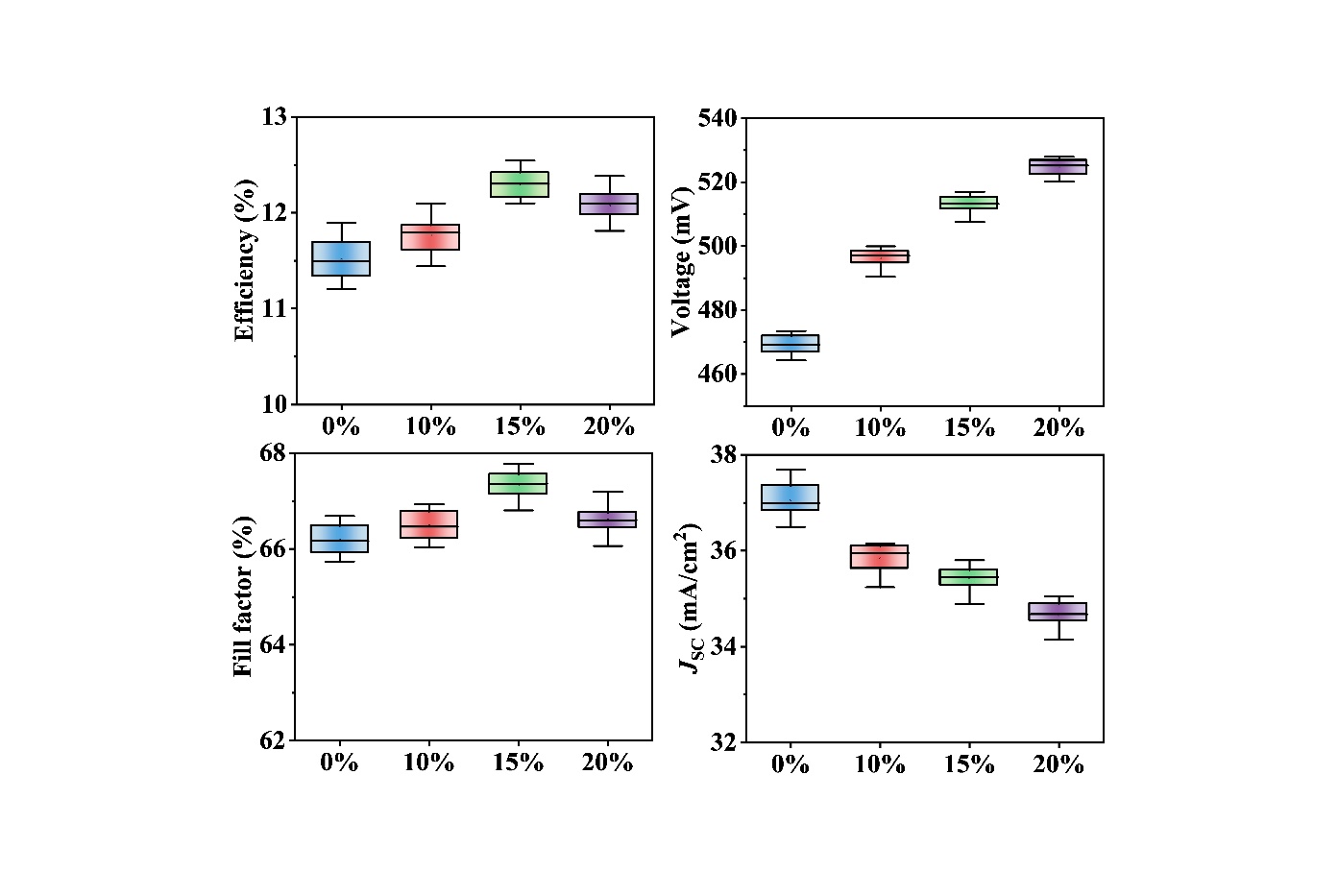


**Fig. S8** Statistical box plots of photovoltaic parameters for the CZTSSe device with different Ag concentrations (0 wt%, 10 wt%, 15 wt%, 20 wt%).


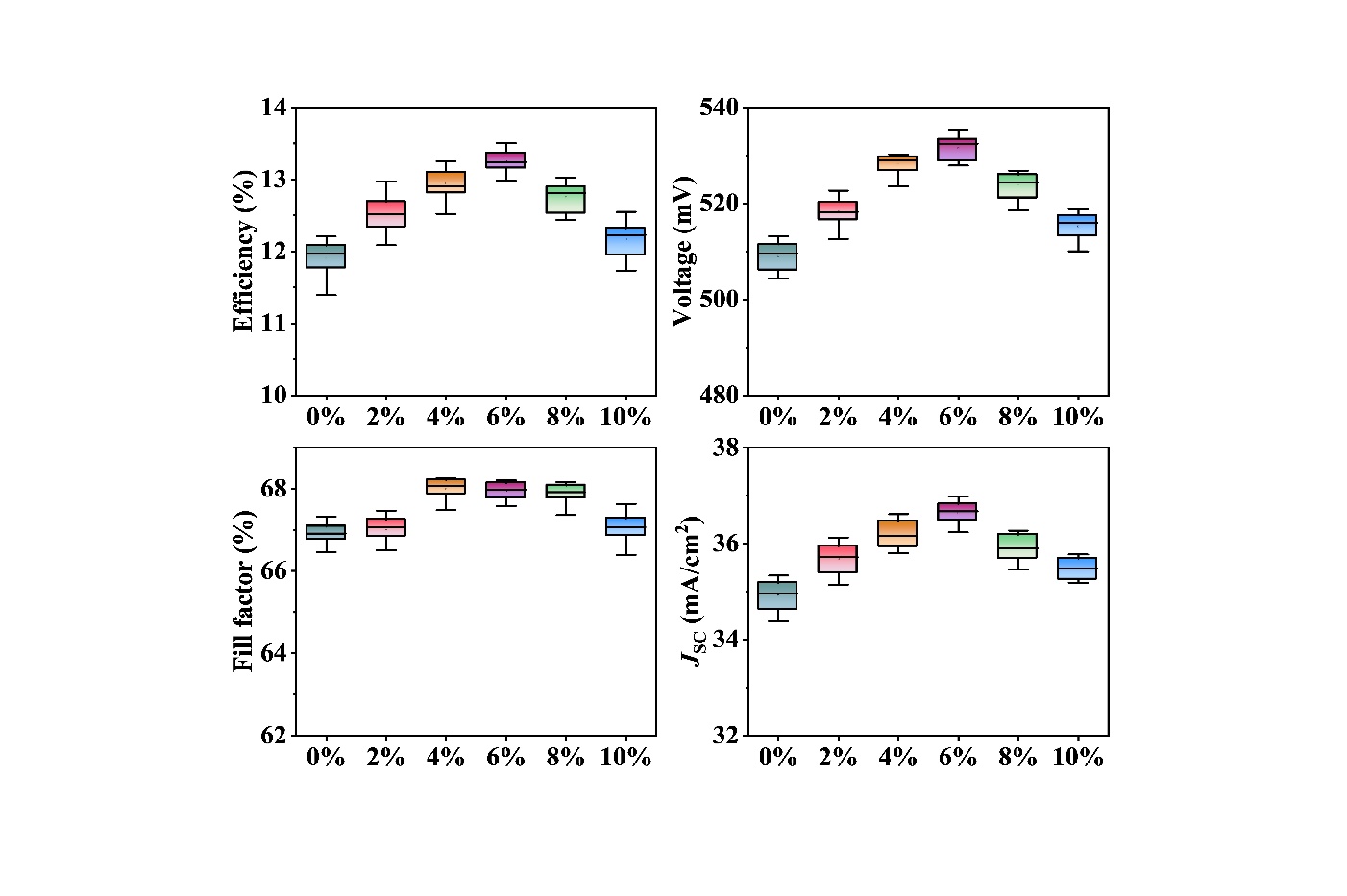


**Fig. S9** Statistical box plots of photovoltaic parameters for the Ag/H co-doped CZTSSe devices with different H_2_ concentrations (0%, 2%, 4%, 6%, 8%, and 10%)


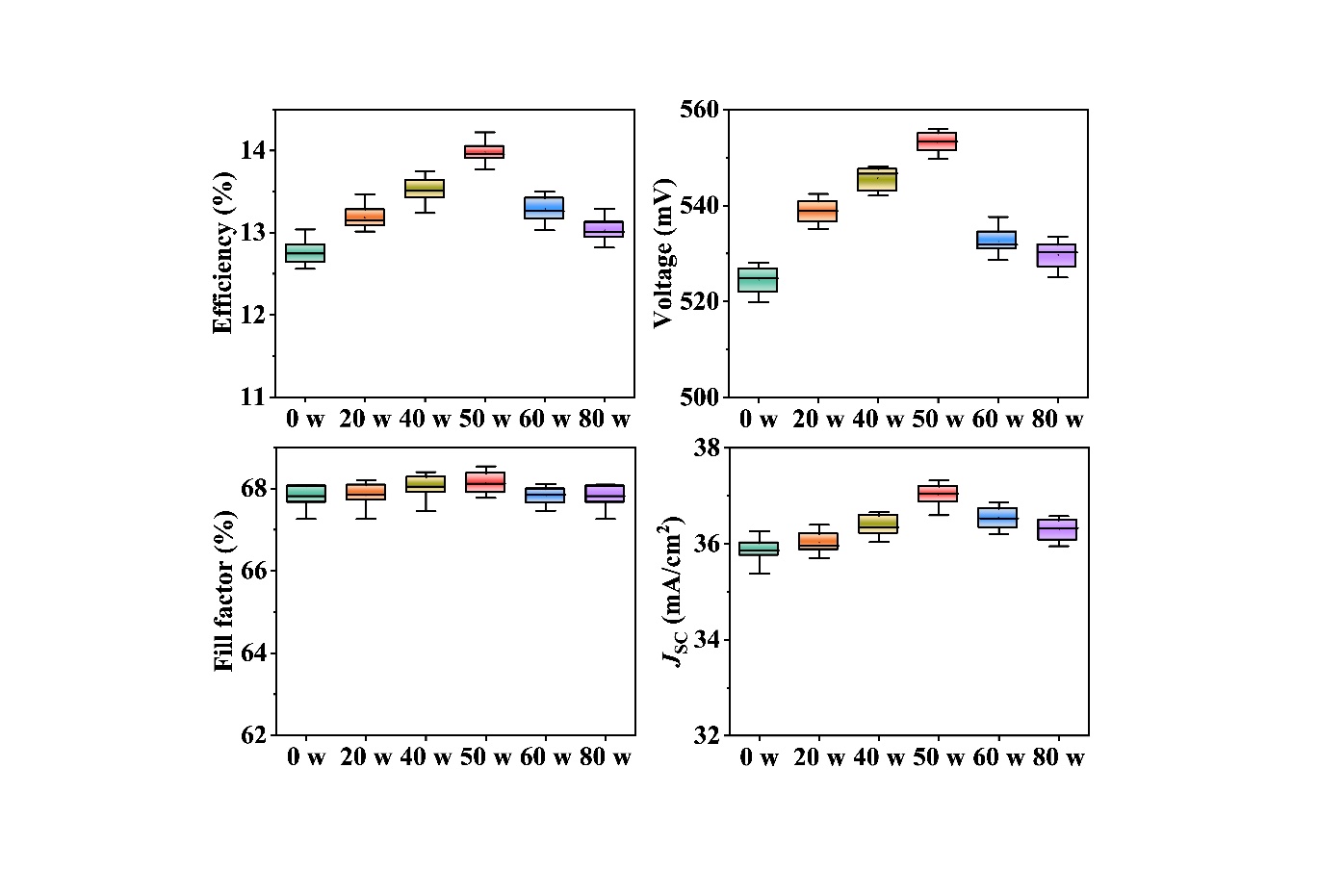


**Fig. S10** Statistical box plots of photovoltaic parameters for the Ag/H co-doped CZTSSe devices with different H-plasma treatment powers (0, 20, 40, 50, 60, and 80 W)


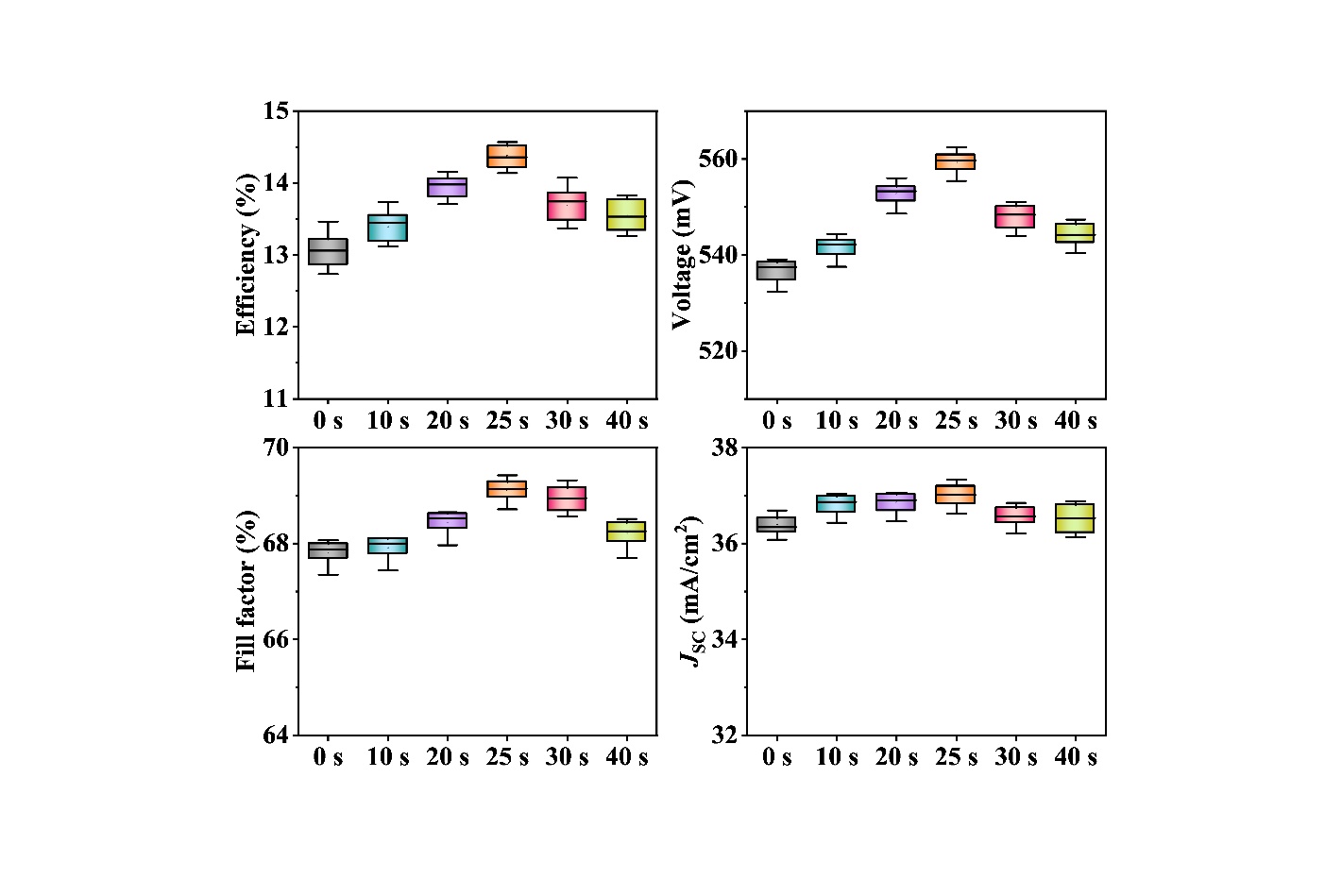


**Fig. S11** Statistical box plots of photovoltaic parameters for the Ag/H co-doped CZTSSe device with different H-plasma treatment times (0, 10, 20, 25, 30, and 40 s)


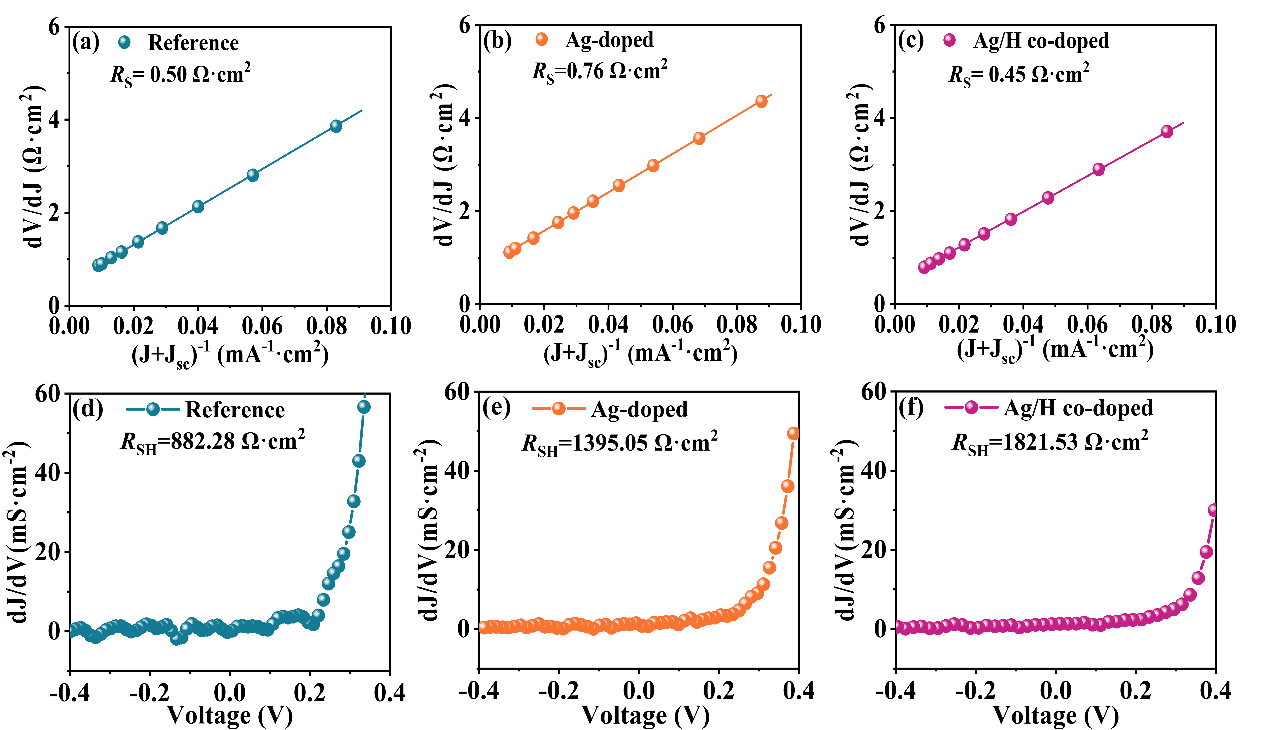


**Fig. S12** Calculation of the diode parameters for the champion Reference, Ag-doped, and Ag/H co-doped CZTSSe devices on the basis of light *J-V* curves: (**a-c**) series resistance *R*_S_, and (**d-f**) shunt resistance *R*_SH_


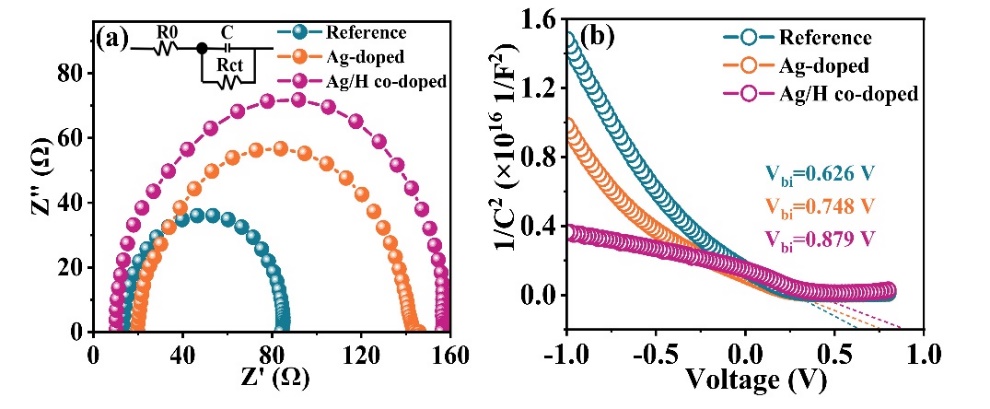


**Fig. S13** **(a)** Impedance plots at a bias of -0.45 V. Inset: the corresponding equivalent circuit. **(b)** 1/C^2^-V curves of Reference, Ag-doped, and Ag/H co-doped CZTSSe devices

**Table S1** The detailed lattice constants of the Reference sample. The average values are calculated from 5 samples with standard deviation

| Sample | | S1 | S2 | S3 | S4 | S5 | Average |
| --- | --- | --- | --- | --- | --- | --- | --- |
| Lattice constant | a(Å) | 5.659 | 5.666 | 5.671 | 5.655 | 5.651 | 5.660±0.007 |
|  | c(Å) | 11.321 | 11.313 | 11.318 | 11.324 | 11.328 | 11.320±0.005 |

**Table S2** The detailed lattice constants of the Ag-doped sample. The average values are calculated from 5 samples with standard deviation

| Sample | | S1 | S2 | S3 | S4 | S5 | Average |
| --- | --- | --- | --- | --- | --- | --- | --- |
| Lattice constant | a(Å) | 5.670 | 5.672 | 5.669 | 5.663 | 5.683 | 5.671±0.006 |
|  | c(Å) | 11.345 | 11.340 | 11.350 | 11.349 | 11.339 | 11.344±0.004 |

**Table S3** The detailed lattice constants of the Ag/H co-doped sample. The average values are calculated from 5 samples with standard deviation

| Sample | | S1 | S2 | S3 | S4 | S5 | Average |
| --- | --- | --- | --- | --- | --- | --- | --- |
| Lattice constant | a(Å) | 5.673 | 5.678 | 5.668 | 5.674 | 5.680 | 5.674±0.004 |
|  | c(Å) | 11.347 | 11.341 | 11.343 | 11.352 | 11.351 | 11.346±0.004 |

**Table S4** Summary of the photovoltaic parameters for the CZTSSe solar cells. The data are the average values calculated from 18 devices with standard deviation

| Sample | PCE [%] | *V*_OC_ [mV] | FF [%] | *J*_SC_ [mA/cm^2^] |
| --- | --- | --- | --- | --- |
| Reference | 11.71±0.17 | 478.44±2.68 | 66.04±0.37 | 36.79±0.35 |
| Ag-doped | 12.30±0.17 | 519.90±2.29 | 68.26±0.33 | 34.74±0.31 |
| Ag/H co-doped | 14.50±0.16 | 561.25±2.21 | 69.47±0.18 | 37.16±0.20 |

**Table S5** Summary of detailed photovoltaic parameters for the CZTSSe solar cells with different Ag-doping levels

| Sample | PCE [%] (%) | *V*_OC_ [mV] | FF [%] | *J*_SC_ [mA/cm^2^] |
| --- | --- | --- | --- | --- |
| 0% | 11.89 | 473.42 | 66.68 | 37.68 |
| 10% | 12.09 | 499.90 | 66.93 | 36.15 |
| 15% | 12.54 | 516.98 | 67.77 | 35.80 |
| 20% | 12.38 | 527.92 | 67.19 | 34.90 |

**Table S6** Summary of the photovoltaic parameters for CZTSSe solar cells with different Ag concentrations (0-20 wt%). The data are the average value calculated from 18 devices with standard deviation

| Sample | PCE [%] (%) | *V*_OC_ [mV] | FF [%] | *J*_SC_ [mA/cm^2^] |
| --- | --- | --- | --- | --- |
| 0% | 11.51±0.22 | 469.22±2.85 | 66.18±0.30 | 37.05±0.33 |
| 10% | 11.76±0.17 | 496.23±2.79 | 66.48±0.29 | 35.84±0.31 |
| 15% | 12.29±0.13 | 513.24±2.66 | 67.32±0.28 | 35.43±0.24 |
| 20% | 12.07±0.15 | 524.73±2.44 | 66.60±0.28 | 34.65±0.24 |

**Table S7** Summary of detailed photovoltaic parameters for the Ag/H co-doped CZTSSe solar cells with different H_2_ concentrations

| Sample | PCE [%] | *V*_OC_ [mV] | FF [%] | *J*_SC_ [mA/cm^2^] |
| --- | --- | --- | --- | --- |
| 0% | 12.20 | 513.18 | 67.32 | 35.33 |
| 2% | 12.73 | 522.68 | 67.47 | 36.12 |
| 4% | 13.25 | 530.25 | 68.25 | 36.61 |
| 6% | 13.50 | 535.37 | 68.20 | 36.97 |
| 8% | 13.02 | 526.85 | 68.16 | 36.27 |
| 10% | 12.55 | 518.81 | 67.62 | 35.77 |

**Table S8** Summary of the photovoltaic parameters for the Ag/H co-doped CZTSSe solar cells with different H_2_ concentrations (0-10%). The data are the average values calculated from 18 devices with standard deviation

| Sample | PCE [%] | *V*_OC_ [mV] | FF [%] | *J*_SC_ [mA/cm^2^] |
| --- | --- | --- | --- | --- |
| 0% | 11.90±0.25 | 509.02±2.84 | 66.92±0.25 | 34.90±0.30 |
| 2% | 12.49±0.23 | 518.28±2.73 | 67.02±0.28 | 35.68±0.31 |
| 4% | 12.94±0.20 | 528.11±2.13 | 67.99±0.25 | 36.17±0.28 |
| 6% | 13.24±0.16 | 531.52±2.38 | 67.93±0.19 | 36.64±0.21 |
| 8% | 12.75±0.19 | 523.72±2.58 | 67.89±0.21 | 35.90±0.26 |
| 10% | 12.17±0.22 | 515.25±2.59 | 67.06±0.34 | 35.47±0.21 |

**Table S9** Summary of detailed photovoltaic parameters for the Ag/H co-doped CZTSSe solar cells with different H-plasma treatment powers

| Sample | PCE [%] (%) | *V*_OC_ [mV] | FF [%] | *J*_SC_ [mA/cm^2^] |
| --- | --- | --- | --- | --- |
| 0 W | 13.03 | 528.09 | 68.08 | 36.26 |
| 20 W | 13.46 | 542.42 | 68.20 | 36.39 |
| 40 W | 13.74 | 548.15 | 68.40 | 36.65 |
| 50 W | 14.22 | 555.92 | 68.54 | 37.32 |
| 60 W | 13.49 | 537.65 | 68.11 | 36.85 |
| 80 W | 13.28 | 533.53 | 68.10 | 36.57 |

**Table S10** Summary of the photovoltaic parameters for the Ag/H co-doped CZTSSe solar cells with different H-plasma treatment powers (0-80 W). The data are the average values calculated from 18 devices with standard deviation

| Sample | PCE [%] | *V*_OC_ [mV] | FF [%] | *J*_SC_ [mA/cm^2^] |
| --- | --- | --- | --- | --- |
| 0 W | 12.75±0.13 | 524.39±2.79 | 67.81±0.23 | 35.85±0.24 |
| 20 W | 13.18±0.13 | 538.72±2.73 | 67.83±0.28 | 36.00±0.22 |
| 40 W | 13.51±0.15 | 545.64±2.26 | 68.02±0.28 | 36.36±0.21 |
| 50 W | 13.97±0.11 | 553.18±2.11 | 68.12±0.25 | 37.01±0.21 |
| 60 W | 13.28±0.14 | 532.50±2.52 | 67.82±0.22 | 36.53±0.21 |
| 80 W | 13.03±0.12 | 529.63±2.64 | 67.81±0.23 | 36.29±0.22 |

**Table S11** Detailed device parameters for the Ag/H co-doped CZTSSe solar cells with various H-plasma treatment times

| Sample | PCE [%] | *V*_OC_ [mV] | FF [%] | *J*_SC_ [mA/cm^2^] |
| --- | --- | --- | --- | --- |
| 0 s | 13.46 | 539.12 | 68.07 | 36.68 |
| 10 s | 13.73 | 544.37 | 68.11 | 37.03 |
| 20 s | 14.16 | 556.00 | 68.65 | 37.06 |
| 25 s | 14.57 | 562.45 | 69.42 | 37.33 |
| 30 s | 14.07 | 551.03 | 69.31 | 36.84 |
| 40 s | 13.83 | 547.38 | 68.51 | 36.87 |

**Table S12** Summary of the photovoltaic parameters for CAZTSSe solar cells with different H-plasma treatment times (0-40 s). The data are the average values calculated from 18 devices with standard deviation

| Sample | PCE [%] | *V*_OC_ [mV] | FF [%] | *J*_SC_ [mA/cm^2^] |
| --- | --- | --- | --- | --- |
| 0 s | 13.04±0.21 | 536.63±2.31 | 67.81±0.21 | 36.37±0.20 |
| 10 s | 13.39±0.20 | 541.57±2.08 | 67.91±0.21 | 36.81±0.19 |
| 20 s | 13.94±0.15 | 552.71±2.12 | 68.44±0.21 | 36.85±0.19 |
| 25 s | 14.37±0.15 | 559.22±2.19 | 69.09±0.20 | 36.99±0.21 |
| 30 s | 13.70±0.21 | 548.09±2.32 | 68.93±0.25 | 36.56±0.20 |
| 40 s | 13.55±0.21 | 544.30±2.37 | 68.20±0.25 | 36.50±0.29 |

**Table S13** Summary of the relevant parameters derived from C-V and DLCP measurements

| Sample | | Reference | Ag-doped | Ag/H co-doped |
| --- | --- | --- | --- | --- |
| N_IT_ (cm^-3^) | 1.20×10^16^ | 8.89×10^15^ | 5.40×10^15^ |  |
| N_BT_ (cm^-3^) | 9.01×10^15^ | 5.17×10^15^ | 1.21×10^15^ |  |
